# External radiation dose reconstruction for settlements near the Semipalatinsk nuclear test site, Kazakhstan, in the international multicenter study: a detailed review and comparative analysis of the initial data

**DOI:** 10.1093/jrr/rraf049

**Published:** 2025-08-30

**Authors:** Valeriy Stepanenko, Sergey Shinkarev, Alexandra Lipikhina, Kazbek Apsalikov, Andrey Kaprin, Sergey Ivanov, Peter Shegay, Evgenia Ostroumova, Viktoria Bogacheva, Yuliya Brait, Kassym Zhumadilov, Masayoshi Yamamoto, Aya Sakaguchi, Satoru Endo, Nariaki Fujimoto, Bernd Grosche, Vladimir Iatsenko, Alla Androsova, Noriyuki Kawano, Masaharu Hoshi

**Affiliations:** A. Tsyb Medical Radiological Research Centre - Branch of the National Medical Research Radiological Centre of the Ministry of Health of the Russian Federation, 4 Koroleva St., Obninsk, Kaluga Region, 2490036, Russian Federation; State Research Center - Burnasyan Federal Medical Biophysical Center of Federal Medical Biological Agency, 46 Zhivopisnaya St., Moscow, 123098, Russian Federation; Scientific Research Institute of Radiation Medicine and Ecology of the non-commercial joint-stock company «Semey Medical University», 258 Gagarin St., Semey, 071407, Republic of Kazakhstan; Scientific Research Institute of Radiation Medicine and Ecology of the non-commercial joint-stock company «Semey Medical University», 258 Gagarin St., Semey, 071407, Republic of Kazakhstan; National Medical Research Radiological Centre of the Ministry of Health of the Russian Federation, 4 Koroleva St., Obninsk, Kaluga Region, 2490036, Russian Federation; Peoples’ Friendship University of Russia, 6 Miklukho-Maklaya St., Moscow, 117198, Russian Federation; P.A. Hertzen Moscow Oncology Research Institute – branch of the National Medical Research Radiological Centre of the Ministry of Health of the Russian Federation, 2nd Botkinsky Drive 3, Moscow, 125284, Russian Federation; A. Tsyb Medical Radiological Research Centre - Branch of the National Medical Research Radiological Centre of the Ministry of Health of the Russian Federation, 4 Koroleva St., Obninsk, Kaluga Region, 2490036, Russian Federation; Peoples’ Friendship University of Russia, 6 Miklukho-Maklaya St., Moscow, 117198, Russian Federation; National Medical Research Radiological Centre of the Ministry of Health of the Russian Federation, 4 Koroleva St., Obninsk, Kaluga Region, 2490036, Russian Federation; Environment and Lifestyle Epidemiology Branch, International Agency for Research on Cancer/WHO, 25 avenue Tony Garnier, Lyon, 69366, France; A. Tsyb Medical Radiological Research Centre - Branch of the National Medical Research Radiological Centre of the Ministry of Health of the Russian Federation, 4 Koroleva St., Obninsk, Kaluga Region, 2490036, Russian Federation; Scientific Research Institute of Radiation Medicine and Ecology of the non-commercial joint-stock company «Semey Medical University», 258 Gagarin St., Semey, 071407, Republic of Kazakhstan; L.N. Gumilyov Eurasian National University, 13 Munaitpasova St., Office 300, Astana, 010008, Republic of Kazakhstan; Low-Level Radioactivity Laboratory, Institute of Nature and Environmental Technology, Kanazawa University, Wakemachi O24, Nomi, Ishikawa, 923-1224, Japan; Institute of Pure and Applied Sciences, University of Tsukuba 1-1-1 Tennodai, Tsukuba, Ibaraki, 305-8577, Japan; Graduate School of Advanced Science and Engineering, Hiroshima University 1-4-1, Kagamiyama, Higashi, Hiroshima, 739-8527, Japan; Research Institute for Radiation Biology and Medicine, 1-2-3, Kasumi, Minami-ku, Hiroshima, 734-8553, Japan; Consultant, formerly: Federal Office for Radiation Protection, Germany, Grasmueckenweg 19, 85356 Freising, Germany; State Research Center - Burnasyan Federal Medical Biophysical Center of Federal Medical Biological Agency, 46 Zhivopisnaya St., Moscow, 123098, Russian Federation; State Research Center - Burnasyan Federal Medical Biophysical Center of Federal Medical Biological Agency, 46 Zhivopisnaya St., Moscow, 123098, Russian Federation; The Center for Peace, Hiroshima University Higashisenda-machi 1-1-89, Naka-ku, Hiroshima, 730-0053, Japan; The Center for Peace, Hiroshima University Higashisenda-machi 1-1-89, Naka-ku, Hiroshima, 730-0053, Japan

**Keywords:** Semipalatinsk nuclear test site, exposure dose rates, ^137^Cs soil contamination, accumulated radiation dose to air, retrospective dosimetry, ESR and TL/OSL dosimetry

## Abstract

Studies on health effects of radiation exposure to residents around the Semipalatinsk nuclear test site (SNTS), Kazakhstan, are necessary for epidemiological assessment of radiation-related health risks after low-dose irradiation. Radiation dose estimates are the principal point for radiation epidemiological studies. These estimates should be based on the most reliable initial data, used for dose estimations. The comparative critical analysis of various available archival and published initial data, namely values of historical exposure dose rates and values of soil contamination by ^137^Cs in considered settlements, including information about dates, times and locations of measurements, was performed with the aim to select most reliable and realistic initial data necessary for estimation of settlement-average accumulated external doses for some settlements located in the vicinity of radioactive clouds’ trajectories related to the most significant tests at the SNTS. Results of estimation of accumulated external radiation dose to air, based on these selected initial data, are presented for 18 settlements. Calculated accumulated external doses were compared with retrospective instrumental dose estimates for settlements, where data on TL/OSL luminescence retrospective dosimetry with quartz-containing samples or electron spin resonance (ESR) dosimetry with tooth enamel were available. Estimation of settlement-average external radiation dose to air is the first important step necessary for the next step—individualized radiation dose estimations among different age, professional and ethnic-specific groups of population lived in the study settlements considering behavior, shielding, location and relocation factors in each population group. This is a subject of future work.

## INTRODUCTION

Studies on radiation exposure and possible health effects in the residents around the Semipalatinsk nuclear test site (SNTS) in Kazakhstan can provide additional scientific evidence on the risks of adverse health outcomes after irradiation in general population [1–9]. Radiation dose estimates are the principal point for radiation epidemiological studies [1]. In a case of radioactive fallout from nuclear tests, exposure dose rates, related to the period of nuclear tests and values of soil contamination by long lived radionuclides, are the main initial data necessary for retrospective accumulated dose estimations [10–32]. On the other hand, available archival and published data on the exposure rates and/or ^137^Cs soil contamination after the tests, which were obtained many years ago, may have different completeness of the information required for dose reconstruction, and sometimes the data reliability is in question. This is the reason, why comparative critical analysis of various available initial data necessary for retrospective dose calculations, namely historical exposure dose rates, values soil contamination by long-lived radionuclides, such as ^137^Cs, including information about dates, times, locations of their measurements and results of instrumental retrospective dosimetry, is an important task of dosimetry support of epidemiological studies on health consequences in relation to the nuclear tests conducted at SNTS [32–62].

Thus, retrospective radiation dose estimates should be based on the most reliable initial data, selected from the comparative analysis of various available archival and published sources on radiological information. In addition, data on TL/OSL luminescence retrospective dosimetry with quartz-containing samples (see for example [14, 63–68]) or electron spin resonance (ESR) dosimetry with tooth enamel (see for example [15, 16, 69–79]) are considered as a useful tool to validate the calculated doses [5].

Here, we present the results of mean external radiation dose to air evaluations for 18 settlements located in the vicinity of radioactive clouds’ trajectories from the most significant nuclear tests at the SNTS. These evaluations were performed in the framework of international multicenter research collaboration using various published and archival initial data necessary for retrospective external dose reconstruction.

It is important to note that external dose estimates are especially important for population health risk assessment, because the values of external doses are much higher compared to internal radiation doses in the population affected by the SNTS nuclear tests, with an exception for thyroid doses from ^131^I internal irradiation [3, 10, 11].

Estimations of settlement-average external radiation dose to air are the first important step necessary for the next step—individualized radiation dose estimations among different age- and ethnic-specific groups of population in the study settlements considering behavior, shielding and location factors in each population group. This is a subject of future work.

## MATERIALS AND METHODS

We performed a comparative analysis of initial data, necessary for retrospective external dose reconstruction, using a pool of initial data published in [4–8, 10, 11, 13, 17–46, 60, 80–83], and archival information from the databases of the of the State Medical Automated Register of the Scientific Research Institute of Radiation Medicine and Ecology (NIIRME), Semey, Kazakhstan [9]. The following initial data are necessary for calculations of settlement-average external dose to air:

- total yield of explosion
- composition of fission material
- height of detonation above ground surface
- height of radioactive cloud
- distance between the place of detonation and study settlement (calculated using GPS data)
- wind speed (averaged over the height of radioactive cloud)
- fallout arrival time
- duration of fallout deposition
- exposure rate in the point of interest
- date of exposure rate measurement
- time dependence of the exposure rate.

For retrospective reconstruction of mean external dose to air in the settlements located near the SNTS, where historical data on exposure dose rates were available, we used the US–Russian Joint Methodology [11, 41, 46, 82] incorporating Russian methodology [10, 40] and the US methodology [46–48]. To convert historical exposure rate units (mR/hour) to absorbed dose rate to air (mGy/hour), a conversion factor of 8.7 × 10^−3^ mGy/mR was used. Accumulated dose to air was calculated for the period from fallout time-of-arrival (TOA) up to one year after the test, because the dose value accumulated in one year constitutes about 98% of the total radiation dose accumulated in the period after the test [10, 11, 32, 40]. The values of TOA for each of considered settlements were estimated according to approach of [48] in a frame of US–Russian methodology [46–48]. A special feature of the joint US–Russian methodology is that the commonly used dependence of dose rate on time *t* after detonation in the form of a power function *t^-n^*, where power ‘*n*’ has various values for different time intervals and different composition of fission materials, is replaced by a 10-term exponential function. This function, describing the dependence of exposure dose rate on time, is applicable for various designs of nuclear devices, including: (i) devices fueled with ^239^Pu but surrounded by steel and lead shielding; (ii) devices fueled by ^238^U; and (iii) devices fueled by pure ^239^Pu, as well as for any time intervals after detonation [46], which makes application of 10-term exponential function more convenient. In addition, the joint methodology does not require separate calculation of contributions to the external radiation dose from the radioactive cloud and from fallout on the ground. This is also convenient, if we consider that in most cases neither the times of fallout start, nor are the times of fallout end known exactly.

In cases where historical dose rates were not available, or were uncertain/questionable, the values of external dose were calculated using available measurement data on long-lived ^137^Cs soil contamination density [4, 12, 13, 17, 18, 20, 21, 25, 32, 39, 50–52, 53–58, 61] in the study settlements using an approach published by Beck *et al.* [61]. This paper presents a model for estimating the external dose rate using ^137^Cs soil contamination density measurements after nuclear tests in or near the trajectories of the radioactive clouds. The approach is based on a usage of published data with the results of exposure dose rates and radionuclide soil contamination data following local fallout from the US and USSR nuclear tests. ^137^Cs soil contamination data are used as a basis for calculating a contribution to the external dose of various radionuclides produced by detonation of different types of fission devices either ^235^U or ^239^Pu or a combination as the fissionable fuel. The fission of this fuel results in creation of a number of fission and activation products as well as a considerable amount of radioactive debris composed of unfissioned fuel (the paper [61] provides a list of 36 corresponding radionuclides). Activity ratios of the considered radionuclides to ^137^Cs activity in a mixture maintaining the same composition as fission products were accounted for the external dose calculation. This model also allows accounting for the fractionation of the composition of radionuclide fallout as a function of the time after explosion and the distance from the trajectory of the radioactive cloud. The value of exposure dose rate to air is estimated at a specified time after the test (24 hours) using the dependence of the dose rate on time approximated by a 10-term exponential function, as described in the US–Russian Joint Methodology [10, 11, 46–48, 82]. In calculations the integration start times of the exposure rates derived from the ^137^Cs deposition density, were accepted as equal to start times, which were used at integration of the available historical dose rates related to the same tests.

There is a similar approach which was used for external dose reconstruction after the nuclear test on 29 August 1949. This approach is described by Imanaka *et al.* [44, 60] for the village of Dolon located near the trajectory of the radioactive cloud related to the 29.08.1949 test. Applying this methodology, external accumulated doses for Dolon village was estimated using available ^137^Cs soil contamination data and calculating the input to the dose of gamma-emitting radionuclides from ^239^Pu fission considering a temporal change in the fission product composition. Thirty radionuclides from the list of fission products were selected, as possible candidates that could significantly contribute to the external accumulated dose from the first SNTS test. Activity ratios of each considered radionuclide to ^137^Cs activity in a mixture maintaining the same composition as fission products were considered. Rare gas species and short half-life radionuclides were excluded considering the fallout arrival time of several hours in the study settlements. Neutron-capture nuclides were not considered because their input to the annual value of external dose is much less compared with the fission products [40, 44]. This approach also accounted for the fractionation of the composition of radionuclide fallout [44, 60].

Similar to [49], we used the following criteria selecting the most reliable published ^137^Cs soil contamination data: (i) soil samples for ^137^Cs measurements had to be taken from the areas without human interventions; and (ii) soil samples taken to a depth 30 cm or more are preferable. But, it should be noted, that even if the above-listed criteria are met, the results of dose estimations, based on the ^137^Cs deposition density, are the values obtained under assumption of ideal environment, where there is no influence of dose reduction factors (for example, such as weathering). It is important to note that the values of ^137^Cs soil contamination density used in our calculations, were corrected by subtracting of contribution from ^137^Cs global fallout, which has been reported at around 500 Bq/m^2^ for Kazakhstan [53, 57].

Available published data obtained by instrumental retrospective dosimetry methods—luminescence retrospective dosimetry using quartz inclusions in the bricks (TL/OSL) or ESR retrospective dosimetry with human tooth enamel [5, 13–16, 62–79] were considered as useful sources of information to validate the calculated external dose values.

## RESULTS

In accordance with [7, 8], the following settlements were selected for mean external dose estimates reconstruction: Akbulak, Belokamenka, Bolshaya Vladimirovka (now Beskaragay), Bodene, Chagan, Cheremushka, Dolon, Kainar, Kanonerka, Karaul, Kaskabulak, Korosteli, Kundidzi (now Zhurekadir), Mostik, Novopokrovka, Sarzhal, Zhetizhar (now Semiyarka), Znamenka (now Kokentau). These settlements were selected based on their proximity to the SNTS, and populations of the settlements are targeted for epidemiological study on possible health effects after the irradiation [7–9]. Results of our external dose estimates to air for each of the 18 study settlements and dose-forming tests, including locations (GPS coordinates) of the settlements and relevant notes, are presented in the Table 1. In addition, and for comparison, Table 1 presents values of external dose to air according to available published data.

**Table 1:** Results of estimations of external dose to air (mGy) for each of 18 study settlements and dose-forming tests—in comparison with available published data

Study settlements with GPS coordinates	Nuclear test date	Mean external dose to air, mGy (this work)	Notes^a^	External dose to air, mGy, according to available published data (with corresponding references)	Notes^b^
Akbulak 49°09′56″ N 77°56′55″ E	24.09.1951	210	Dose estimation on the base of ^137^Cs soil contamination density data.	820 [83]	[83] - Dose estimation on the base of exposure rate data^c^
Belokamenka 50°33′31″ N 79°36′05″ E	29.08.1949	<1 (0.4–0.5)	Dose estimation on the base of exposure rate data.	0.9 [82]	[82] - Dose assessment based on isolines with values of external dose to air ​​drawn on archival map in the vicinity of settlement
	29.07.1955	<1 (0.08–0.1)	Dose estimation on the base of exposure rate data.	NA^d^	
	07.08.1962	<1 (0.35–0.56)	Dose estimation on the base of exposure rate data.	NA^d^	
Bolshaya Vladimirovka (now Beskaragay) 50°53′09″ N 79°28′58″ E	29.08.1949	<1.5 (1–1.4)	Dose estimation on the base of exposure rate data. Not in contradiction with the results of TL/OSL measurements in quartz-containing samples of bricks within the measurements uncertainty (see Supplementary Table 4)	1.8 [82]	[82] - Dose assessment based on isolines with values of external dose to air ​​drawn on archival map in the vicinity of settlement
	29.07.1955	≤1 (0.8–1)	Dose estimations on the base of exposure rate data.	NA^d^	
Bodene 50°37′02″ N 79°06′32″ E	29.08.1949	260 (200–320)	Dose estimation on the base of exposure rate data. Consistent with the dose estimations on the base of ^137^Cs soil contamination density data. Agrees with rough estimates based on the results of ESR retrospective dosimetry method (see Supplementary Table 5).	200 [82]	[82] - Dose assessment based on isolines with values of external dose to air ​​drawn on archival map in the vicinity of settlement
Chagan 50°37′16″ N 79°15′09″ E	29.08.1949	220 (190–240)	Dose estimation on the base of exposure rate data. Consistent with the dose estimations from ^137^Cs soil contamination density data.	NA^d^	
	29.07.1955	<1 (0.7–0.9)	Dose estimations on the base of exposure rate data.	NA^d^	
	15.01.1965	70	Dose estimation on the base of exposure rate data.	NA	
Cheremushka 50°38′38″ N 79°03′30″ E	29.08.1949	430	Dose estimation on the base of exposure rate data. Not in contradiction with dose estimations on the base of ^137^Cs soil contamination density data and with estimation of dose to air based on the results of ESR retrospective dosimetry method (see Supplementary Table 7).	1700 [82] 2970 [83]	[34] - Dose assessment based on values of external dose to air related to the centerline of radioactive cloud’s trajectory drawn on archival map; [83] - Dose estimation on the base of exposure rate data ^***)^
	07.08.1962	≤1.6 (0.16–1.6)	Dose estimations on the base of exposure rate data.	NA^d^	
Dolon 50°39′33″ N 79°18′25″ E	29.08.1949	500 (350–650)	Dose estimation on the base of exposure rate data with accounting for ^137^Cs soil contamination data. Not in contradiction with estimates of dose to air based on results of TL/OSL measurements in quartz-containing samples from bricks, and on the results of ESR retrospective dosimetry using tooth enamel samples (see Supplementary Table 8).	600 (350–910) [44, 60] 1950 [82] 1950 [83]	[44, 60] - Dose estimation based on ^137^Cs soil contamination data; [34] -Dose assessment based on values of external dose to air related to the centerline of radioactive cloud’s trajectory drawn on archival map; [83] - Dose estimation on the base of exposure rate data ^***)^
	29.07.1955	<1 (0.4–0.6)	Dose estimation on the base of exposure rate data.	NA^d^	
	07.08.1962	≤1.5 (0.1–1.5)	Dose estimation on the base of exposure rate data.	NA^d^	
Kainar 49°12′02″ N 77°23′05″ E	24.09.1951	210 (75–350)	Dose estimation on the base of exposure rate data. Not in contradiction with the dose estimation based on ^137^Cs soil contamination density data.	114–870 [82] 360 [83]	[82] - Dose assessment based on isolines with values of external dose to air ​​drawn on archival map in the vicinity of settlement; [83]- Dose estimation on the base of exposure rate data ^***)^
	05.10.1954	11 (0.5–22)	Dose estimation on the base of exposure rate data.	NA^d^	
	02.08.1955	22 (13–31)	Dose estimation on the base of exposure rate data.	NA^d^	
Kanonerka 50°43′24″ N 79°41′24″ E	29.08.1949	210 (130–310)	Dose estimation on the base of exposure rate data. Consistent with the results of TL/OSL measurements in quartz-containing samples of bricks. It is not in contradiction with the dose estimations based on ^137^Cs soil contamination density data, as well as with rough estimate of dose to air based on the results of ESR retrospective dosimetry method (see Supplementary Table 10).	500 [82] 350 [83]	[82] - Dose assessment based on isolines with values of external dose to air ​​drawn on archival map in the vicinity of settlement [83] - Dose estimation on the base of exposure rate data ^***)^
	29.07.1955	≤1 (0.7–1)	Dose estimation on the base of exposure rate data.	NA^d^	
	07.08.1962	≤1 (0.14–1)	Dose estimation on the base of exposure rate data.	NA^d^	
Karaaul 48°56′38″ N 79°15′44″ E	12.08.1953	660 (380–940)	Dose estimation on the base of exposure rate data. Not in contradiction with external dose estimates based on ^137^Cs soil contamination data. When recalculation dose to air to external dose to the residents of Karaaul, it will be necessary to consider the exact timing of residents’ temporary evacuation from the settlement.	890–1300 [82] 120–380 [57] 1150 [83]	[82] - Dose assessment based on values of external dose to air related to the centerline of radioactive cloud’s trajectory drawn on archival map; [57] - Dose estimates based on ^137^Cs soil contamination data; [83] - Dose estimation on the base of exposure rate data ^***)^
Kaskabulak 49°33′52″ N 79°52′18″ E	30.10.1954	40 (17–60)	Dose estimation on the base of exposure rate data. Agrees with the external dose estimations based on ^137^Cs soil contamination data.	NA^d^	
Korosteli 51°02′46″ N 80°59′52″ E	29.08.1949	38 (34–45)	Dose estimation on the base of exposure rate data.	NA^d^	
Kundizdi (now Zhurekadir) 48°32′54″ N 79°37′46″ E	30.10.1954	46 (39–53)	Dose estimation on the base of exposure rate data.	NA^d^	
Mostik 50°41′15″ N 79°06′44″ E	29.08.1949	170 (110–230)	Dose estimation on the base of exposure rate data with accounting for dose estimates based on ^137^Cs soil contamination data. It agrees with rough estimate of dose to air based on the results of ESR retrospective dosimetry method (see Supplementary Table 15).	180 [82] 170 [83]	[82] - Dose assessment based on isolines with values of external dose to air ​​drawn on archival map in the vicinity of settlement; [83] - Dose estimation on the base of exposure rate data ^***)^
	07.08.1962	<1 (0.15–0.24)	Dose estimation on the base of exposure rate data.	NA^d^	
Novopokrovka 50°40′10″ N 80°27′47″ E	29.08.1949	0.26	Dose estimation on the base of exposure rate data. Agrees with external dose estimates based on ^137^Cs soil contamination data.	NA^d^	
	29.07.1955	<1 (0.69–0.93)		NA^d^	
	07.08.1962	<1 (0.51–0.94)		NA^d^	
Sarzhal 49°36′00″ N 78°44′18″ E	12.08.1953	850 (460–1250)	Dose estimation on the base of exposure rate data. Not in contradiction with external dose estimates based on ^137^Cs soil contamination data. When recalculation dose to air to external dose to the residents of Sarzhal, it will be necessary to consider the exact timing of residents’ temporary evacuation from the settlement.	930 [82] 210–530 [57] 2090 [83]	[82] - Dose assessment based on isolines with values of external dose to air ​​drawn on archival map in the vicinity of settlement; [57] - Dose estimates based on ^137^Cs soil contamination data; [83] - Dose estimation on the base of exposure rate data ^***)^
	30.10.1954	35 (30–40)	Dose estimation on the base of exposure rate data.	NA^d^	
Zhetizhar (now Semiyarka) 50°53′41″ N 78°19′30″ E	07.08.1962	3 (0.25–5.6)	Dose estimation on the base of exposure rate data.	NA^d^	
Znamenka 50°04′42″ N 79°34′56″ E	12.08.1953	26 (13–39)	Dose estimation on the base of exposure rate data. Agrees with the external dose estimates based on ^137^Cs soil contamination data.	NA^d^	
	30.10.1954	<1 (0.22–0.96)	Dose estimation on the base of exposure rate data.	NA^d^	
	16.03.1956	22 (16–28)		NA^d^	
	24.08.1956	67 (23–110)		80 [82] 120 [83]	[82] - Dose assessment based on isolines with values of external dose to air ​​drawn on archival map in the vicinity of the settlement; [83] - Dose estimation on the base of exposure rate data ^***)^
	07.08.1962	≤1 (0.28–1)		NA^d^	
	25.09.1962	5 (0.7–9.4)		NA^d^	
	15.01.1965	62		NA^d^	

^a^Additional comments are available in ‘Supplement’ (Tables ST 2–19).

^b^Notes to values of external dose to air, which are presented in cited available publications.

^c^The exact origin of the data on the exposure dose rates, that were used to calculate the external doses to air, and locations of dose rate measurement in relation to the settlement, are not specified with sufficient details in this publication for each test and for each settlement considered.

^d^The data are not available.

Total number of nuclear tests, which were conducted at the Semipalatinsk Test Site is equal to 456, including 111 above-ground tests (86 in air and 25 on surface) [27, 33–38]. The reviews of these tests are presented in a number of publications (see, for example [8–11, 21, 22, 82]).

**Fig. 1:**
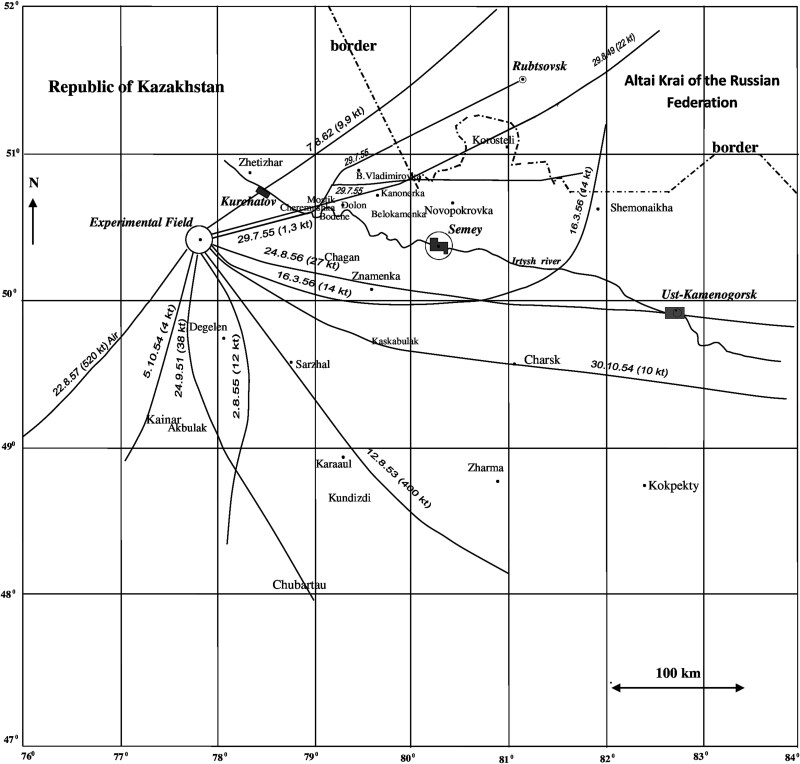
Schematic trajectories of the radioactive clouds related to the most significant tests at the SNTS with indication of considered settlements.

Trajectories of the radioactive clouds related to ‘most significant’ 11 tests are shown in Fig. 1 [82].

It should be noted here, that according to publications [10, 11, 17, 40, 41, 82], the criterion used for selection of the ‘most significant’ nuclear test, which are shown in Fig. 1, was as follows: the test is considered as the ‘most significant’ if the lifetime effective dose to an adult person constantly living during the year following the nuclear explosion at the point of maximum off-site exposure rate would exceed 5 mSv. This criterion was considered as conservative as far as it assumes that a person constantly remains at the point with the maximum level of radiation exposure power, for example, directly on the axis of the ‘trace’. It is important to note that here and further in this paper, including the Supplement, the meaning of the term ‘trace’ is the following: ‘the pattern of fallout downwind from the point of detonation’, which is similar to a term by Gordeev *et al.* [40, 41], Beck *et al.* [61]. Nevertheless, this criterion corresponds to the Radiation Safety Standards, in which the maximum level of annual effective dose for the population was stated as 1 mSv per year on average over any five consecutive years, but not more than 5 mSv per year [84, 85].

As noted above, the trajectories of the radioactive clouds, which are presented in Fig. 1, are related to the tests considered as ‘most significant’ according to [10, 82]. It is important to note here, that due to high level of conservatism, the effective doses, used as criteria for selection of ‘most significant’ tests nuclear events, do not reflect actual radiation doses to the population. Therefore, the more realistic dose estimates should be performed in order to use these estimates in epidemiological studies. Selection of ‘most significant’ events from all nuclear tests, conducted at the SNTS, allows the specialists to concentrate their efforts on the analysis of radiological conditions and dose assessment from those events that might have resulted in substantial exposure to the populations [82].

More detailed data regarding initial information necessary for estimations of settlement-average external dose to air, with the results of our estimations of mean external dose to air based on these data, and with additional comments/discussion are presented in the Supplementary Tables 1–19 (ST 1–19).

Supplementary Table 1 (ST1) provides the list of 18 settlements included in the study with the corresponding location coordinates (GPS data). Available information on dates, heights, yields and locations of the tests, which radioactive fallout were considered as possible sources of external irradiation of the population, is provided in Table 1 (data from [24, 26, 27, 32–38] and archival information from the database of NIIRME State Registry [9, 17, 32] are presented). Supplementary Tables 2–19 (ST 2–19) contain comments with comparisons, explanatory of selection of the most reliable archival/published initial data, which were used for external dose reconstruction, along with the results of our estimations of external dose to air based on these data for each of 18 study settlements. Calculated accumulated external doses were compared with retrospective instrumental dose estimates for settlements, where data on TL/OSL luminescence retrospective dosimetry with quartz-containing samples or Electron Spin Resonance (ESR) dosimetry with tooth enamel were available.

## CONCLUSION AND DISCUSSION

In this paper we have gathered and critically reviewed available archival and published initial data, namely historical exposure dose rates, values soil contamination by ^137^Cs, including information about dates, times and locations of their measurements, and results of instrumental retrospective dosimetry, necessary for estimation of accumulated external dose in air among 18 settlements located in the vicinity of the Semipalatinsk nuclear test site. The populations of 18 considered settlements are targeted for an epidemiological study on possible health effects after the irradiation [7–9].

Results of estimation of accumulated external radiation dose to air, based on selected most reliable initial data, are presented above, in the Table 1 of the main text. External dose estimates to air were performed for each of 18 study settlements and for 13 nuclear tests, which are related to fallout in and near these settlements. According to [10, 82], eleven of these tests were conservatively ranked as the ‘most significant’. More detailed data regarding selection of initial information necessary for estimations of settlement-average external dose to air, including results of estimations of mean external dose to air based on these data, and with corresponding comments, are presented in the Supplementary Tables 1–19. Complimentary information and discussion related to our estimations of external dose values is included in the text of the supplementary material.

Table 1 presents the values of external dose to air according to available published data as well—in order to compare with our dose estimates. These comparisons were performed for those settlements, for which the relevant information was found in available literature. Additional information and discussion related to the comparisons between values of accumulated external radiation doses in the air, calculated by authors, with previously published values of external radiation doses in the air is included in the text of the supplementary material.

To validate the values of external radiation doses, which were calculated using archival dose rate data, the available published results of dose estimations obtained by instrumental methods of retrospective dosimetry (TL/OSL dosimetry using quartz inclusions [86, 87] and ESR dosimetry using tooth enamel) were accounted for.

Dose calculations based on archival dose rate data were compared, where possible, with retrospective dose calculations based on ^137^Cs soil contamination data. As a result, values of external radiation dose are presented as point dose estimates with an indication, where possible, of dose ranges. The corresponding comments are presented in the notes to Table 1 in the main text of the article, as well as in the Tables 1–19 in the supplementary material. (including special comments to these Tables).

Additional comments regarding peculiarities of different calculational and instrumental retrospective dosimetry methods were included in the supplementary material.

Selection of the most reliable input data followed by estimations of mean external dose to air is the first important step necessary for the next step—individualized radiation dose estimations among different age, professional and ethnic-specific groups of population lived in the study settlements considering behavior, shielding, location and relocation factors in each population group. This is the subject of our future work.

## Supplementary Material

JRRS_D_25_00036_R1_Suppl_Table_1_Revised_No_Hig_rraf049

JRRS_D_25_00036_R1_Suppl_Table_2_revised_No_Hig_rraf049

JRRS_D_25_00036_R1_Supplementary_Table_3_Revised_rraf049

JRRS_D_25_00036_R1_Suppl_Table_4_Revised_No_Hig_rraf049

JRRS_D_25_00036_R1_Suppl_Table_5_Revised_No_Hig_rraf049

JRRS_D_25_00036_R1_Supplementary_Table_6_revised_rraf049

JRRS_D_25_00036_R1_Suppl_Table_7_Revised_No_Hig_rraf049

JRRS_D_25_00036_R1_Suppl_Table_8_revised_No_Hig_rraf049

JRRS_D_25_00036_R1_Suppl_Table_9_Revised_No_Hig_rraf049

JRRS_D_25_00036_R1_Suppl_Table_10_Revised_No_Hig_rraf049

JRRS_D_25_00036_R1_Supplementary_Table_11_revised_rraf049

JRRS_D_25_00036_R1_Supplementary_Table_12_Revised_rraf049

JRRS_D_25_00036_R1_Suppl_Table_13_Revised_No_Hig_rraf049

JRRS_D_25_00036_R1_Supplementary_Table_14_revised_rraf049

JRRS_D_25_00036_R1_Suppl_Table_15_Revised_No_Hig_rraf049

JRRS_D_25_00036_R1_Supplementary_Table_16_Revised_rraf049

JRRS_D_25_00036_R1_Supplementary_Table_17_Revised_rraf049

JRRS_D_25_00036_R1_Supplementary_Table_18_Revised_rraf049

JRRS_D_25_00036_R1_Supplementary_Table_19_revised_rraf049

8_JRRS_D_25_00036_R1_Supplement_revised_No_Highlight_2025_rraf049

## Data Availability

All authors are ready to provide data upon request.
