# Supplementary material for: External radiation dose reconstruction for settlements near the Semipalatinsk nuclear test site, Kazakhstan, in the international multicenter study: a detailed review and comparative analysis of the initial data
Source: J Radiat Res. 2025 Aug 30;66(5):496–508. doi: 10.1093/jrr/rraf049 (PMC12460053; doi:10.1093/jrr/rraf049)
Supplement: JRRS_D_25_00036_R1_Suppl_Table_1_Revised_No_Hig_rraf049 [file jrrs_d_25_00036_r1_suppl_table_1_revised_no_hig_rraf049.docx]

Supplementary Table 1 (ST 1). List of 18 study settlements with available information on the dates, heights, yields, settlements’ coordinates and locations of the tests, for which the radioactive fallout were considered as possible sources of external irradiation of the population (data from [25, 27, 28, 33-39], and from database of NIIRME State Registry [10, 18, 33] are presented - see List of references in the main part of the paper).

| No | Study settlements with GPS coordinates | Date of nuclear test | Explosion height, yield, location | Height of radioactive cloud, km | Distance to SNTS, km | Average wind speed, km/h |
| --- | --- | --- | --- | --- | --- | --- |
| 1 | Akbulak  [49°09′56″ N 77°36′55″ E](https://ru.wikipedia.org/wiki/%D0%90%D0%BA%D0%B1%D1%83%D0%BB%D0%B0%D0%BA_(%D0%B3%D0%BE%D1%80%D0%BE%D0%B4%D1%81%D0%BA%D0%B0%D1%8F_%D0%B0%D0%B4%D0%BC%D0%B8%D0%BD%D0%B8%D1%81%D1%82%D1%80%D0%B0%D1%86%D0%B8%D1%8F_%D0%A1%D0%B5%D0%BC%D0%B5%D1%8F)#/maplink/1) | 24.09.1951 | 30m,  38kT,  50^0^23’18’’N,  77^0^53’30’’E | 11.4 | 165 | 26.4 |
| 2 | Belokamenka  [50°33′31″ N 79°36′05″ E](https://ru.wikipedia.org/wiki/%D0%91%D0%B5%D0%BB%D0%BE%D0%BA%D0%B0%D0%BC%D0%B5%D0%BD%D0%BA%D0%B0_(%D0%91%D0%B5%D1%81%D0%BA%D0%B0%D1%80%D0%B0%D0%B3%D0%B0%D0%B9%D1%81%D0%BA%D0%B8%D0%B9_%D1%80%D0%B0%D0%B9%D0%BE%D0%BD)#/maplink/1) | 29.07.1955 | on the surface,  1.3 kT  50^0^ N, 78^0^ E | 5.2 | 122 | 42 |
|  |  | 07.08.1962 | on the surface,  9.9 kT  50^0^17’18’’N,  77^0^40’54’’E | 8.9 |  | 10 |
|  |  | 29.08.1949 | 30m,  22 kT,  50^0^23’18’’N,  77^0^53’30’’E | 10,4 |  | 47 |
| 3 | Bolshaya Vladimirovka (now Beskaragay)  [50°53′09″ N 79°28′58″ E](https://ru.wikipedia.org/wiki/%D0%91%D0%B5%D1%81%D0%BA%D0%B0%D1%80%D0%B0%D0%B3%D0%B0%D0%B9_(%D0%92%D0%BE%D1%81%D1%82%D0%BE%D1%87%D0%BD%D0%BE-%D0%9A%D0%B0%D0%B7%D0%B0%D1%85%D1%81%D1%82%D0%B0%D0%BD%D1%81%D0%BA%D0%B0%D1%8F_%D0%BE%D0%B1%D0%BB%D0%B0%D1%81%D1%82%D1%8C)#/maplink/1) | 29.07.1955 | on the surface,  1.3 kT  50^0^ N, 78^0^ E | 5.2 | 134 | 42 |
|  |  | 29.08.1949 | 30m,  22 kT,  50^0^23’18’’N,  77^0^53’30’’E | 10,4 |  | 47 |
| 4 | Bodene  [50°37′02″ N 79°06′32″ E](https://ru.wikipedia.org/wiki/%D0%91%D0%BE%D0%B4%D0%B5%D0%BD%D0%B5_(%D0%92%D0%BE%D1%81%D1%82%D0%BE%D1%87%D0%BD%D0%BE-%D0%9A%D0%B0%D0%B7%D0%B0%D1%85%D1%81%D1%82%D0%B0%D0%BD%D1%81%D0%BA%D0%B0%D1%8F_%D0%BE%D0%B1%D0%BB%D0%B0%D1%81%D1%82%D1%8C)#/maplink/1) | 29.08.1949 | 30m,  22 kT,  50^0^23’18’’N,  77^0^53’30’’E | 10,4 | 103 | 47 |
| 5 | Chagan  [50°37′16″ N 79°15′09″ E](https://ru.wikipedia.org/wiki/%D0%A7%D0%B0%D0%B3%D0%B0%D0%BD_(%D0%BF%D0%BE%D1%81%D1%91%D0%BB%D0%BE%D0%BA)#/maplink/1) | 29.07.1955 | on the surface,  1.3 kT,  50^0^ N, 78^0^ E | 5.2 | 70 | 42 |
|  |  | 26.11.1962 | on the surface,  0.031 kT,  50^0^08’32N,  78^0^04’18’’E | - |  | - |
|  |  | 15.01.1965 | Under ground level - with excavation,  -178m,  140kT,  49.94^0^ N, 79.01^0^ E | 4.8 | 75 | 40 |
|  |  | 29.08.1949 | 30m,  22 kT,  50^0^23’18’’N,  77^0^53’30’’E | 10,4 | 70 | 47 |
| 6 | Cheremushka  [50°38′38″ N 79°03′30″ E](https://ru.wikipedia.org/wiki/%D0%A7%D0%B5%D1%80%D1%91%D0%BC%D1%83%D1%88%D0%BA%D0%B0_(%D0%91%D0%B5%D1%81%D0%BA%D0%B0%D1%80%D0%B0%D0%B3%D0%B0%D0%B9%D1%81%D0%BA%D0%B8%D0%B9_%D1%80%D0%B0%D0%B9%D0%BE%D0%BD)#/maplink/1) | 07.08.1962 | on the surface,  9.9 kT,  50^0^17’18’’N  77^0^40’54’’E | 8.9 | 76 | 10 |
|  |  | 29.08.1949 | 30m,  22 kT,  50^0^23’18’’N  77^0^53’30’’E | 10,4 | 76 | 47 |
| 7 | Dolon  [50°39′33″ N 79°18′25″ E](https://ru.wikipedia.org/wiki/%D0%94%D0%BE%D0%BB%D0%BE%D0%BD%D1%8C#/maplink/1) | 29.07.1955 | on the surface,  1.3 kT,  50.0^0^ N,  78.0^0^ E | 5.2 | 118 | 42 |
|  |  | 07.08.1962 | on the surface,  9.9 kT,  50^0^17’18’’N,  77^0^40’54’’E | 8.9 | 118 | 10 |
|  |  | 29.08.1949 | 30m,  22 kT,  50^0^23’18’’N,  77^0^53’30’’E | 10.4 | 113 | 47 |
| 8 | Kainar  [49°12′02″ N 77°23′05″ E](https://ru.wikipedia.org/wiki/%D0%9A%D0%B0%D0%B9%D0%BD%D0%B0%D1%80_(%D0%B3%D0%BE%D1%80%D0%BE%D0%B4%D1%81%D0%BA%D0%B0%D1%8F_%D0%B0%D0%B4%D0%BC%D0%B8%D0%BD%D0%B8%D1%81%D1%82%D1%80%D0%B0%D1%86%D0%B8%D1%8F_%D0%A1%D0%B5%D0%BC%D0%B5%D1%8F)#/maplink/1) | 24.09.1951 | 30m,  38kT,  50^0^23’18’’N  77^0^53’30’’E | 11.4 | 138 | 26.4 |
|  |  | 05.10.1954 | on the surface,  4 kT,  50^0^ N, 78^0^ E | 7.3 | 139 | 43.3 |
|  |  | 02.08.1955 | on the surface,  12 kT,  50^0^ N, 78^0^ E | 9.3 | 140 | 36.6 |
| 9 | Kanonerka  [50°43′24″ N 79°41′24″ E](https://ru.wikipedia.org/wiki/%D0%9A%D0%B0%D0%BD%D0%BE%D0%BD%D0%B5%D1%80%D0%BA%D0%B0_(%D0%92%D0%BE%D1%81%D1%82%D0%BE%D1%87%D0%BD%D0%BE-%D0%9A%D0%B0%D0%B7%D0%B0%D1%85%D1%81%D1%82%D0%B0%D0%BD%D1%81%D0%BA%D0%B0%D1%8F_%D0%BE%D0%B1%D0%BB%D0%B0%D1%81%D1%82%D1%8C)#/maplink/1) | 29.08.1949 | 30m,  22 kT,  50^0^23’18’’N,  77^0^53’30’’E | 10.4 | 142 | 47 |
|  |  | 29.07.1955 | on the surface,  1.3 kT,  50.0^0^ N,78.0^0^ E | 5.2 | 135 | 42 |
|  |  | 07.08.1962 | on the surface,  9.9 kT,  50^0^17’18’’N,  77^0^40’54’’E | 8.9 | 135 | 10 |
| 10 | Karaul  [48°56′38″ N 79°15′44″ E](https://ru.wikipedia.org/wiki/%D0%9A%D0%B0%D1%80%D0%B0%D0%B0%D1%83%D0%BB_(%D0%90%D0%B1%D0%B0%D0%B9%D1%81%D0%BA%D0%B8%D0%B9_%D1%80%D0%B0%D0%B9%D0%BE%D0%BD)#/maplink/1) | 12.08.1953 | 30m,  400 kT,  50^0^23’18’’N,  77^0^53’30’’E | 15.8 | 188 | 64.6 |
| 11 | Kaskabulak  [49°33′52″ N 79°52′18″ E](https://ru.wikipedia.org/wiki/%D0%9A%D0%B0%D1%81%D0%BA%D0%B0%D0%B1%D1%83%D0%BB%D0%B0%D0%BA_(%D0%92%D0%BE%D1%81%D1%82%D0%BE%D1%87%D0%BD%D0%BE-%D0%9A%D0%B0%D0%B7%D0%B0%D1%85%D1%81%D1%82%D0%B0%D0%BD%D1%81%D0%BA%D0%B0%D1%8F_%D0%BE%D0%B1%D0%BB%D0%B0%D1%81%D1%82%D1%8C)#/maplink/1) | 30.10.1954 | 55 m,  10 kT,  50^0^27’10’’N,  77^0^49’16’’E | 9 | 160 | 32.9 |
| 12 | Korosteli  [51°02′46″ N 80°59′52″ E](https://ru.wikipedia.org/wiki/%D0%9A%D0%BE%D1%80%D0%BE%D1%81%D1%82%D0%B5%D0%BB%D0%B8_(%D0%92%D0%BE%D1%81%D1%82%D0%BE%D1%87%D0%BD%D0%BE-%D0%9A%D0%B0%D0%B7%D0%B0%D1%85%D1%81%D1%82%D0%B0%D0%BD%D1%81%D0%BA%D0%B0%D1%8F_%D0%BE%D0%B1%D0%BB%D0%B0%D1%81%D1%82%D1%8C)#/maplink/1) | 29.08.1949 | 30m,  22 kT  50^0^23’18’’N,  77^0^53’30’’E | 10.4 | 239 | 47 |
| 13 | Kundidzi  (now Zhurekadir)  [48°32′54″ N 79°37′46″ E](https://ru.wikipedia.org/wiki/%D0%96%D1%83%D1%80%D0%B5%D0%BA%D0%B0%D0%B4%D1%8B%D1%80#/maplink/1) | 30.10.1954 | 55 m,  10 kT,  50^0^27’10’’N  77^0^49’16’’E | 9 | 183 | 32.9 |
| 14 | Mostik  [50°41′15″ N 79°06′44″ E](https://ru.wikipedia.org/wiki/%D0%9C%D0%BE%D1%81%D1%82%D0%B8%D0%BA_(%D1%81%D0%B5%D0%BB%D0%BE)#/maplink/1) | 07.08.1962 | on the surface,  9.9 kT,  50^0^17’18’’N,  77^0^40’54’’E | 8.9 | 90 | 10 |
|  |  | 29.08.1949 | 30m,  22 kT,  50^0^23’18’’N,  77^0^53’30’’E | 10.4 | 90 | 47 |
| 15 | Novopokrovka  [50°40′10″ N 80°27′47″ E](https://ru.wikipedia.org/wiki/%D0%9D%D0%BE%D0%B2%D0%BE%D0%BF%D0%BE%D0%BA%D1%80%D0%BE%D0%B2%D0%BA%D0%B0_(%D0%92%D0%BE%D1%81%D1%82%D0%BE%D1%87%D0%BD%D0%BE-%D0%9A%D0%B0%D0%B7%D0%B0%D1%85%D1%81%D1%82%D0%B0%D0%BD%D1%81%D0%BA%D0%B0%D1%8F_%D0%BE%D0%B1%D0%BB%D0%B0%D1%81%D1%82%D1%8C)#/maplink/1) | 29.08.1949 | 30m,  22 kT,  50^0^23’18’’N  77^0^53’30’’E | 10.4 | 186 | 47 |
|  |  | 29.07.1955 | on the surface,  1.3 kT  50.0^0^ N,78.0^0^ E | 5.2 | 180 | 42 |
|  |  | 07.08.1962 | on the surface,  9.9 kT,  50^0^17’18’’N,  77^0^40’54’’E | 8.9 | 180 | 10 |
| 16 | Sarzhal  [49°36′00″ N 78°44′18″ E](https://ru.wikipedia.org/wiki/%D0%A1%D0%B0%D1%80%D1%8B%D0%B6%D0%B0%D0%BB_(%D0%92%D0%BE%D1%81%D1%82%D0%BE%D1%87%D0%BD%D0%BE-%D0%9A%D0%B0%D0%B7%D0%B0%D1%85%D1%81%D1%82%D0%B0%D0%BD%D1%81%D0%BA%D0%B0%D1%8F_%D0%BE%D0%B1%D0%BB%D0%B0%D1%81%D1%82%D1%8C)#/maplink/1) | 30.10.1954 | 55m,  10kT,  50^0^27’10’’N,  77^0^49’16’’E | 9 | 151 | 32.9 |
|  |  | 12.08.1953 | 30m,  400 kT,  50^0^23’18’’N,  77^0^53’30’’E | 15.8 | 110 | 64.6 |
| 17 | Semiyarka  (now Zhetizhar)  [50°53′41″ N 78°19′30″ E](https://ru-wiki.ru/wiki/%D0%A1%D0%BB%D1%83%D0%B6%D0%B5%D0%B1%D0%BD%D0%B0%D1%8F:Map/14/50.8948361/78.3250083/ru) | 07.08.1962 | on the surface,  9.9 kT,  50^0^17’18’’N,  77^0^40’54’’E | 8.9 | 62 | 10 |
| 18 | Znamenka  (now Kokentau)  [50°04′42″ N 79°34′56″](https://ru.wikipedia.org/wiki/%D0%9A%D0%BE%D0%BA%D0%B5%D0%BD%D1%82%D0%B0%D1%83#/maplink/1)E | 12.08.1953 | 30m,  400 kT,  50^0^23’18’’N,  77^0^53’30’’E | 15.8 | 110 | 64.6 |
|  |  | 30.10.1954 | 55m,  10kT,  50^0^27’10’’N,  77^0^49’16’’E | 9 | 125 | 32.9 |
|  |  | 16.03.1956 | on the surface,  14 kT,  50^0^17’18’’N,  77^0^40’54’’E | 9.6 | 125 | 39 |
|  |  | 07.08.1962 | on the surface,  9.9 kT,  50^0^17’18’’N  77^0^40’54’’E | 8.9 | 125 | 10 |
|  |  | 25.09.1962 | on the surface,  7 kT,  50.0^0^ N,78.0^0^ E | 8.3 | 125 | 32.4 |
|  |  | 15.01.1965 | Under ground level, with excavation,  -178m,  140kT,  49.94^0^ N, 79.01^0^ E | 4.8 | 125 | 40 |
|  |  | 24.08.1956 | 93 m,  27 kT,  50.00 N  78.00 E | 10.8 | 125 | 71.2 |
