# Supplementary material for: External radiation dose reconstruction for settlements near the Semipalatinsk nuclear test site, Kazakhstan, in the international multicenter study: a detailed review and comparative analysis of the initial data
Source: J Radiat Res. 2025 Aug 30;66(5):496–508. doi: 10.1093/jrr/rraf049 (PMC12460053; doi:10.1093/jrr/rraf049)
Supplement: JRRS_D_25_00036_R1_Suppl_Table_2_revised_No_Hig_rraf049 [file jrrs_d_25_00036_r1_suppl_table_2_revised_no_hig_rraf049.docx]

| Supplementary Table 2 (ST 2). Settlement Akbulak. Available exposure dose rate data, ^137^Cs soil contamination density, and calculated external doses to air based on these data^*)^ (see List of references in the main part of the paper). | | | | | | |
| --- | --- | --- | --- | --- | --- | --- |
| Date of explosion | Time related to exposure rate estimation, H+h, h | Exposure  rate | Units | Time of fallout arrival, h | Reference | Calculated dose to air based on exposure rate data,  mGy |
| 24.09.1951 | 24 | 3.14 | R/h | 7.5 | [43] | 3240 |
| 24.09.1951 | 24 | 881.01 | mR/h |  | [33] | 910 |
| 24.09.1951 | 42 | 1.6 | R/h |  | [43] | 3140 |
| ^*)^ Comments to Supplementary Table 2:   - Exposure rates data are available for only one test related to Akbulak. - It is not clear, what is the origin of exposure rate values - direct measurements or results of recalculation from the unknown time of measurements to the time indicated in the Supplementary Table 2 (24 h and 42 h). Moreover, exposure rates, indicated in the Supplementary Table 2, are not consistent with the external dose estimates based on the results of ^137^Cs soil contamination density measurements in Akbulak (see comments below). - For the Akbulak doses derived from archival exposure rate data of 910-3200 mGy, are much higher (by factor of 4-15) than our estimate of external dose to air equal to 210 mGv derived from the ^137^Cs soil contamination density equal to 6700 Bq×m^-2^ in 1989 [26]. - It is interesting to compare external dose estimates for Akbulak settlement with the dose estimates in Kainar settlement; because Akbulak is located relatively close to the settlement of Kainar (distance between these two settlements is about 17 km). - Levels of ^137^Cs deposition in both settlements seemed to be in the same range. For Akbulak, the level of the ^137^Cs soil contamination density of 6700 Bq×m^-2^ at 1989 [26] resulted in our estimate of external dose to air equal to 210 mGv. For Kainar village, measured levels of the ^137^Cs soil contamination densities were equal to 4100 Bq×m^-2^ in 1989 [26] and 1740±1290 Bq/m^2^ in 2007-2012 [58], which resulted in estimates of external doses to air equal to 130 mGy and 90±67 mGy, respectively (see Supplementary Table 9). The uncertainties of the average values ​​given here correspond to two standard deviations (± 2SD). - Moreover, external dose estimates derived from the archival exposure rate data for Kainar are in the range of 75 mGy - 350 mGy, which is not in contradiction with external dose estimates derived from the ^137^Cs soil contamination density in this settlement of 90±67 mGy (see Supplementary Table 9). The uncertainties of the average values ​​given here correspond to two standard deviations (± 2SD). - Possible reasons for the discrepancy between the dose estimates based on exposure rates and based on ^137^Cs contamination data for Akbulak seems to be as follows: 1) exposure rate data related to Akbulak do not reflect exposure rate measurements done in the settlement, and these archival data might be a result of theoretical calculations related the trajectory of radioactive cloud outside Akbulak settlement (distance from Akblak to supposed centerline of the trajectory of radioactive cloud is about 38 km), or 2) these exposure rate data related to Akbulak are mistaken due to possible misprints in the data base’ records for about one order of magnintude. | | | | | | |
| Conclusion: Summing up all the data and considerations above, the estimated settlement-average external dose to air in Akbulak based on ^137^Cs soil contamination density data, is 210 mGy, which is not in contradiction with the range of external dose in Kainar village based on archival exposure rate data - 75 mGy - 350 mGy. | | | | | | |
