# Supplementary material for: External radiation dose reconstruction for settlements near the Semipalatinsk nuclear test site, Kazakhstan, in the international multicenter study: a detailed review and comparative analysis of the initial data
Source: J Radiat Res. 2025 Aug 30;66(5):496–508. doi: 10.1093/jrr/rraf049 (PMC12460053; doi:10.1093/jrr/rraf049)
Supplement: JRRS_D_25_00036_R1_Supplementary_Table_3_Revised_rraf049 [file jrrs_d_25_00036_r1_supplementary_table_3_revised_rraf049.docx]

Supplementary Table 3 (ST 3). Settlement Belokamenka. Available exposure dose rate data and calculated external doses to air based on these data^*)^ (see List of references in the main part of the paper).

| Date of explosion | Time related to exposure rate estimation,  H+h, h | Exposure  rate | Units | Time of fallout arrival, h | Reference | Calculated  dose to air, mGy |
| --- | --- | --- | --- | --- | --- | --- |
| 29.08.1949 | 24 | 0.385 | mR/h | 3.1 | [29, 42, 43, 81] | 0.5 |
| 29.08.1949 | 173 | 0.036 | mR/h |  | [43] | 0.4 |
| 29.08.1949 | 173 | 0.036 | mR/h |  | [19, 33] |  |
| 29.08.1949 | 173 | 0.036 | mR/h |  | [44] |  |
| 29.08.1949 | 173 | 0.036 | mR/h |  | [26, 81] |  |
| 29.07.1955 | 3 | 0.001 | R/h | 3.5 | [29, 32, 42] | 0.08 |
| 29.07.1955 | 24 | 0.08 | mR/h |  | [18, 33] | 0.1 |
| 29.07.1955 | NA^&^ | 0.001 | R/h |  | [20] | NA |
| 07.08.1962 | 216 | 0.045 | mR/h | 14.6 | [29] | 0.41 |
| 07.08.1962 | 264 | 0.03 | mR/h |  | [32] | 0.35 |
| 07.08.1962 | 504 | 0.02 | mR/h |  | [33] | 0.56 |

| ^*)^ Comments to Supplementary Table 3:  - NA^&^ - Time related to exposure rate estimation is not available in publications.   - Exposure rates data for three tests related to fallout in and around Belokamenka were identified. - According to the available exposure rates data and our estimates, each of the three tests resulted in the external dose to air less than 1 mGy for Belokamenka. - No data on ^137^Cs contemporary measurements in and around Belokamenka were found.   Conclusion: For Belokamenka, external dose to air from each considered test was less than 1 mGy: 0.4-0.5 mGy, 0.08-0.1 mGy and 0.35-0.56 mGy for tests 29.08.1949, 29.07.1955 and 07.08.1962, respectively |
| --- |
