# Supplementary material for: External radiation dose reconstruction for settlements near the Semipalatinsk nuclear test site, Kazakhstan, in the international multicenter study: a detailed review and comparative analysis of the initial data
Source: J Radiat Res. 2025 Aug 30;66(5):496–508. doi: 10.1093/jrr/rraf049 (PMC12460053; doi:10.1093/jrr/rraf049)
Supplement: JRRS_D_25_00036_R1_Suppl_Table_4_Revised_No_Hig_rraf049 [file jrrs_d_25_00036_r1_suppl_table_4_revised_no_hig_rraf049.docx]

Supplementary Table 4 (ST 4). Settlement Bolshaya Vladimirovka (now Beskaragay). Available exposure dose rate data and calculated external doses to air based on these data^*)^ (see List of references in the main part of the paper).

| Date of explosion | Time related to exposure rate estimation, H+h, h | Exposure rate | Units | Time of fallout arrival, h | Reference | Calculated dose to air, mGy |
| --- | --- | --- | --- | --- | --- | --- |
| 29.08.1949 | 24 | 1.14 | mR/h | 3.4 | [42, 43, 29] | 1.4 |
| 29.08.1949 | 24 | 1.14 | mR/h |  | [40, 10, 31] |  |
| 29.08.1949 | 173 | 0.1 | mR/h |  | [42] | 1.0 |
| 29.07.1955 | 3 | 0.01 | R/h | 3.8 | [29, 42, 32] | 0.8 |
| 29.07.1955 | 24 | 0.83 | mR/h |  | [33,18] | 1.0 |
| 29.07.1955 | - | 0.01 | R/h |  | [40.20] | - |

| *) Comments to Supplementary Table 4:   - Data on exposure rates for two tests related to fallout in and around Bolshaya Vladimirovka (Beskaragay) were identified. - It is not clear, what is the origin of exposure rate data, direct measurements or the results of recalculation from the real time of measurements to the time shown in the Supplementary Table 4. - All available data on exposure rate measurements (or estimates) show low values of external dose to air, about 1 mGy. - There are no data on ^137^Cs soil contamination density measurements related to Bolshaya Vladimirovka (Beskaragay) settlement. - Accumulated dose to air in the settlement of Bolshaya Vladimirovka (Beskaragay) based on TL/OSL-measurements in quartz-containing samples of bricks [15] was estimated as <50 mGy. Results of these measurements do not exceed the uncertainty of TL/OSL measurements (±50 mGy) [15]. The uncertainties of the average values ​​given here correspond to two standard deviations (± 2SD).   Conclusion: Use of archival exposure rate data, with accounting for results of TL/OSL measurements in bricks resulted in external dose estimation for Bolshaya Vladimirovka settlement as follows: the dose range 1-1.4 mGy for test 29.08.1949, and the dose range 0.8-1.0 mGy for test 29.07.1955. |
| --- |
