# Supplementary material for: External radiation dose reconstruction for settlements near the Semipalatinsk nuclear test site, Kazakhstan, in the international multicenter study: a detailed review and comparative analysis of the initial data
Source: J Radiat Res. 2025 Aug 30;66(5):496–508. doi: 10.1093/jrr/rraf049 (PMC12460053; doi:10.1093/jrr/rraf049)
Supplement: JRRS_D_25_00036_R1_Suppl_Table_5_Revised_No_Hig_rraf049 [file jrrs_d_25_00036_r1_suppl_table_5_revised_no_hig_rraf049.docx]

Supplementary Table 5 (ST 5). Settlement Bodene. Available exposure dose rate data and calculated external doses to air based on these data^*)^ (see List of references in the main part of the paper).

| Date of explosion | Time related to exposure rate estimation, H+h, h | Exposure rate | Units | Time of fallout  arrival, h | Reference | Calculated  dose to air, mGy |
| --- | --- | --- | --- | --- | --- | --- |
| 29.08.1949 | 1.124 | 9.24 | R/h | 2.6 | [19, 29] | 200 |
| 29.08.1949 | 24 | 9.24 | R/h |  | [43] | 12700 |
| 29.08.1949 | 24 | 235 | mR/h |  | [33] | 320 |
| 29.08.1949 | 24 | 0.235 | R/h |  | [18] |  |

| ^*)^ Comments to Supplementary Table 5:   - Only one test was identified in relation to fallout in and around Bodene. - It is not clear, what is the origin of exposure rate data, direct measurements or the results of recalculation from the real time of measurements to the time shown in the Supplmentary Table 5. - Based on two exposure rates values [18, 19, 29, 33] for fallout in and around Bodene, we estimated dose to air equal to 200 mGy and 320 mGy. One record with the exposure rate of 9.24 R/h assigned to the time H+24h, which is resulted in unusual dose value (12700 mGy or 12.7Gy), seems to be mistaken due to possible misprint of time value in the publication [43]. - The values of the available ^137^Cs soil deposition density data related to Bodene are in the range 1097-6979 Bq×m^-2^ with average value of 3560 Bq×m^-2^ [57, 58] that resulted in our estimate of dose to air equal to 350 mGy (range 110-690 mGy), which is not in contradiction with the dose estimates, based on the exposure rate data. - The available results of the assessment of individual doses by the instrumental EPR method of retrospective dosimetry on samples of tooth enamel in nine people from the settlement of Bodene show that the average value of the external radiation dose in this group of residents is 74 mGy (range 23-120 mGy) [16, 50, 74-76]. These nine people lived in the settlement of Bodene for at least one year from the time of the test. Interpretation of the ESR data needs consideration for shielding, behavior, location and migration factors for the inhabitants. These factors are reducing ESR dose in relation to dose to air. According to [5, 14] the mean value of the combination of these factors is 0.28 ± 0.068 for Kazakhstan village. The uncertainties of the average values ​​given here correspond to two standard deviations (± 2SD). As a result, the rough estimate of dose to air based on ESR data is 74 mGy/0.28 = 260 mGy, which is not in contradiction with the dose estimates, based on the exposure rate data. Conclusion: Summing up all the data and considerations above, the estimated settlement-average dose to air based on exposure rate values in Bodene is in the range from 200-320 mGy (mean value 260 mGy). |
| --- |
