# Supplementary material for: External radiation dose reconstruction for settlements near the Semipalatinsk nuclear test site, Kazakhstan, in the international multicenter study: a detailed review and comparative analysis of the initial data
Source: J Radiat Res. 2025 Aug 30;66(5):496–508. doi: 10.1093/jrr/rraf049 (PMC12460053; doi:10.1093/jrr/rraf049)
Supplement: JRRS_D_25_00036_R1_Supplementary_Table_6_revised_rraf049 [file jrrs_d_25_00036_r1_supplementary_table_6_revised_rraf049.docx]

Supplementary Table 6 (ST 6). Settlement Chagan. Available exposure dose rate data and calculated external doses to air based on these data^*)^ (see List of references in the main part of the paper).

| Date of explosion | Time related to exposure rate estimation, H+h, h | Exposure rate | Units | Time of fallout arrival, h | Reference | Calculated dose to air, mGy |
| --- | --- | --- | --- | --- | --- | --- |
| 29.08.1949 | 3 | 1.85 | R/h | 1.8 | [29, 42, 43, 81] | 190 |
| 29.08.1949 | 3 | 1.85 | R/h |  | [31] |  |
| 29.08.1949 | 24 | 0.153 | R/h |  | [33] | 240 |
| 29.07.1955 | 3 | 7.16 | mR/h | 2.0 | [29, 32, 42] | 0.67 |
| 29.07.1955 | 3 | 7.16 | mR/h |  | [31] |  |
| 29.07.1955 | 24 | 0.59 | mR/h |  | [19, 40] | 0.90 |
| 26.11.1962 | 3 | 0.8 | mR/h | - | [31] | - |
| 26.11.1962 | 3 | 0.84 | mR/h |  | [33] | - |
| 26.11.1962 | 3 | 0.84 | mR/h |  | [19, 40] | - |
| 26.11.1962 | 24 | 0.07 | mR/h |  | [18, 33] | - |
| 15.01.1965 | 24 | 0.05 | R/h | 2.3 | [32, 33] | 73 |

| ^*)^ Comments to Supplementary Table 6:   - Four tests were identified in relation to fallout in and around Chagan. - Available archival exposure rate estimates for Chagan for the test on 29.08.1949 provided external dose estimations in the range of 190-240 mGy. - Only one dose rate value was available for the test on 15.01.1965, which provides the settlement-average dose equal to 73 mGy. - Two archival exposure rates estimations were available for Chagan for the test on 29.07.1955. For this test the settlements-average external dose of less than 1 mGy was estimated. - For the test on 26.11.1962, there was no information on average wind speed. However, if we assume wind speed to be of about 38 km/h (as the mean value for the other tests), then the estimated settlement-average dose to air is about 0.1 mGy. - Available measurements of ^137^Cs soil contamination density assigned to Chagan settlement are in the range of 1056-2755 Bq×m^-2^ in 1995 [58]. These values of ^137^Cs soil contamination density correspond to external dose estimations in the range of 77-200 mGy, which is not in contradiction with the dose estimates based on the available archival exposure rates after the tests on 15.01.1965 and 29.08.1949.   Conclusion: Summing up all the data and considerations above, the estimated settlement-average dose to air in Chagan is 220 mGy with the range of 190-240 mGy after the test on 29.08.1949, and 70 mGy after the test on 15.01.1965. For the test on 29.07.1955, the estimated range of external dose to air is 0.7-0.9 mGy. |
| --- |
