# Supplementary material for: External radiation dose reconstruction for settlements near the Semipalatinsk nuclear test site, Kazakhstan, in the international multicenter study: a detailed review and comparative analysis of the initial data
Source: J Radiat Res. 2025 Aug 30;66(5):496–508. doi: 10.1093/jrr/rraf049 (PMC12460053; doi:10.1093/jrr/rraf049)
Supplement: JRRS_D_25_00036_R1_Suppl_Table_7_Revised_No_Hig_rraf049 [file jrrs_d_25_00036_r1_suppl_table_7_revised_no_hig_rraf049.docx]

Supplementary Table 7 (ST 7). Settlement Cheremuska. Available exposure dose rate data and calculated external doses to air based on these data^*)^ (see List of references in the main part of the paper).

| Date of explosion | Time related to exposure rate estimation, H+h, h | Exposure rate | Units | Time of fallout arrival, h | Reference | Calculated  dose to air, mGy |
| --- | --- | --- | --- | --- | --- | --- |
| 29.08.1949 | 24 | 1.3 | R/h | 1.9 | [29, 40, 43, 81] | 2 000 |
| 29.08.1949 | 24 | 385-1300 | mR/h |  | [19] | 590-2000 |
| 29.08.1949 | 173 | 0.012 | R/h |  | [18, 33, 81] | 140 |
| 29.08.1949 | 173 | 12 | mR/h |  | [18] |  |
| 10.09.1956 | - | 0.001 | R/h | - | [4,26] | - |
| 17.01.1958 | - | 0.002 | R/h | - | [44] | - |
| 09.09.1961 | - | 0.001 | R/h | - | [43] | - |
| 07.08.1962 | 2 | 5 | mR/h | 9.1 | [29,42,40] | 0.16 |
| 07.08.1962 | 24 | 0.253 | mR/h |  | [20] | 0.25 |
| 07.08.1962 | 24 | 1.64 | mR/h |  | [33] | 1.6 |
| 07.08.1962 | - | 0.005 | R/h |  | [32] | - |

| ^*)^ Comments to Supplementary Table 7:   - Five tests were identified related to fallout in and around Cheremushka. - For three tests (10.09.1956, 17.01.1958, 09.09.1961) there was no information on the time of measurements. Available values of the exposure rate measurements are very low. Indirectly they show low possible estimates of the settlement-average dose to air, which are comparable to annual radiation background level (about 1 mGy/y). - For the test on 07.08.1962, available exposure rate data for Cheremushka are consistent and show low values of external dose to air (range 0.16-1.6 mGy). - Only one test on 29.08.1949 was significant for dose reconstruction in the residents of Cheremushka. - According to the available information, the actual measurements of exposure rate along the trajectory of radioactive cloud from the first nuclear test at the SNTS were performed about a week after the date of explosion [33, 81]. Thus, the exposure rate data at H+24 h, in fact, are estimates only (not measurements). While the exposure rate data at H+173 h are probably to be the actual measurements. So, the exposure rate data at H+173 h are preferable compared to H+24 h, which results in 140 mGy estimate of the settlement-average dose to air. - Available measurements of ^137^Cs soil contamination density assigned to Cheremushka of 660-8357 Bq×m^-2^ in 2002 [57, 60], is about 25% less than the range of ^137^Cs soil contamination density assigned to in and around Dolon of 768-10314 Bq×m^-2^ in 2002. The range of the ^137^Cs soil contamination density corresponds to the estimates of dose to air in the range from 63 mGy to 800 mGy (with mean value of 430 mGy). - Results of individual dose estimations using instrumental ESR method of retrospective dosimetry with human tooth enamel samples show the dose value averaged among three inhabitants of the settlement, equal to 114 mGy (range 54-180 mGy) [16, 50, 74-76]. These three people lived in the settlement of Bodene for at least one year from the time of the test. Interpretation of the ESR data needs consideration for shielding, behavior, location and migration factors for the inhabitants. These factors are reducing ESR dose in relation to dose to air. According to [5, 14] the mean value of the combination of these factors is 0.28 ± 0.068 for Kazakhstan village. The uncertainties of the average values ​​given here correspond to two standard deviations (± 2SD). As a result, the rough estimate dose to air based on ESR data is 114 mGy/0.28 = 410 mGy (range 200-640 mGy), which is not in contradiction with the dose estimates, based on ^137^Cs soil contamination data.   Conclusion:  Summing up all the data and considerations above, the estimated settlement-average dose to air in Cheremushka based om ^137^Cs soil contamination data is 430 mGy (range 63-800 mGy) for the test on 29.08.1949, which is not in contradicition with dose estimates based on ESR dosimetry data. It is close to dose value for Dolon village (500 mGy). The distance between Cheremushka and Dolon villages is equal to 28 km. Both villages are located close to the trajectory of radioactive cloud (about 2-3 km) [14, 50].  For the test on 07.08.1962, the estimated range of external dose to air is 0.16-1.6 mGy. |
| --- |
