# Supplementary material for: External radiation dose reconstruction for settlements near the Semipalatinsk nuclear test site, Kazakhstan, in the international multicenter study: a detailed review and comparative analysis of the initial data
Source: J Radiat Res. 2025 Aug 30;66(5):496–508. doi: 10.1093/jrr/rraf049 (PMC12460053; doi:10.1093/jrr/rraf049)
Supplement: JRRS_D_25_00036_R1_Suppl_Table_8_revised_No_Hig_rraf049 [file jrrs_d_25_00036_r1_suppl_table_8_revised_no_hig_rraf049.docx]

Supplementary Table 8 (ST 8). Settlement Dolon. Available dose rate data and calculated external doses to air based on these data^*)^ (see List of references in the main part of the paper).

| Date of explosion | Time related to exposure rate estimation,  H+h, h | | Exposure  rate | Units | Time of fallout arrival, h | Reference  43,82 | Calculated dose to air,  mGy |
| --- | --- | --- | --- | --- | --- | --- | --- |
| 29.08.1949 | 3 | | 15.5 | R/h | 2.9 | [29, 32, 42, 43, 81, 82] | 510^&^ |
| 29.08.1949 | 24 | | 1.1-1.3 | R/h |  | [33] | 550-650^&^ |
| 29.08.1949 | 24 | | 1.273 | R/h |  | [18] | 630^&^ |
| 29.08.1949 | 24 | | 1.041 | R/h |  | [19] | 520^&^ |
| 29.08.1949 | 24 | | 1.15 | R/h |  | [11] | 570^&^ |
| 29.08.1949 | 172 | | 0.119 | R/h |  | [43] | 450^&^ |
| 29.08.1949 | 173 | | 0.098 | R/h |  | [33] | 370^&^ |
| 29.08.1949 | 173 | | 0.12 | R/h |  | [44] | 450^&^ |
| 29.08.1949 | 173 | | 100 | mR/h |  | [33] | 380^&^ |
| 29.07.1955 | 3 | | 0.0045 | R/h | 3.4 | [29, 32] | 0.4 |
| 29.07.1955 | 3 | | 5 | mR/h |  | [40] | 0.4 |
| 29.07.1955 | 24 | | 0.412 | mR/h |  | [33] | 0.5 |
| 29.07.1955 | 24 | | 0.37 | mR/h |  | [18] | 0.5 |
| 29.07.1955 | 24 | | 0.5 | mR/h |  | [32] | 0.6 |
| 07.08.1962 | 2 | | 5 | mR/h | 14.2 | [29, 32, 42] | 0.1 |
| 07.08.1962 | 24 | | 1.64 | mR/h |  | [33] | 1.5 |
| 07.08.1962 | 24 | | 1 | mR/h |  | [43] | 0.9 |
| 07.08.1962 | 216 | | 0.025 | mR/h |  | [18] | 0.2 |
| 07.08.1962 | 504 | | 0.01 | mR/h |  | [18] | 0.3 |
|  | | ^&^Note: Assuming that the ratio of exposure rate in Dolon to the exposure rate at the trajectory of radioactive cloud is equal to 0.37±0.024. This ratio was derived as follows: a) on the dose value related to the trajectory of radioactive cloud [11, 18, 19, 33, 43, 44, 81, 82], and b) dose in the settlement [14, 45, 50, 89 ] calculated in accordance with the gradient of dose rate [81] and with gradient of radioactive contamination [14, 55] in the direction from the trajectory of radioactive cloud into the village of Dolon. The village of Dolon is located close to the trajectory of radioactive cloud (about 2 km) [14]. | | | | | |

| ^*)^ Comments to Supplementary Table 8:   - Settlement Dolon is the most studied settlement regarding the retrospective dose estimations as far as a special international study was performed in this settlement to compare different retrospective dosimetry methods in relation to the SNTS [5, 6]. - Three tests were identified in relation to fallout in and around Dolon. - For two tests (29.07.1955 and 07.08.1962) the available exposure rate data for Dolon are consistent and show low values of the settlement-average dose to air, less than 1 mGy. - Only one test on 29.08.1949 was significant for external dose estimation in Dolon. - The available results of exposure rate measurements in September-October 1949 (test on 29.08.1949) are related to the trajectory of radioactive cloud, but not to Dolon village. The external dose to air in Dolon village was calculated as it explaned in the footnote above. As a result, the settlement-average dose to air following the test on 29.08.1949 based on the exposure rate data was estimated to be equal to 500 mGy with the range of 370-650 mGy. - Available measurements of ^137^Cs soil contaminationn density assigned to Dolon village provides average value of 5870 (range 4070-7670 Bq×m^-2^) [13, 26, 44, 57]. According to recommendations in the relevant methodological guidelines [18], the settlement-average radiation dose was estimated on the base of averaged values of ^137^Cs soil contamination densities measured in soil samples taken from different sampling locations. In the case of Dolon village, the value of average soil contamination density is 5870 Bq×m^-2^ (range 4070-7670 Bq×m^-2^). The corresponding estimate of the average external radiation dose in Dolon gives the value of 500 mGy (range 350-650 mGy). The external radiation dose value equal to 600 mGy (range 350-910 mGy) [45,89] in Dolon village was independently estimated by Imanaka et al [45, 89] using data of ^137^Cs soil contamination density. - The estimates of settlement-average dose to air based on TL or OSL measurements of quartz-containing samples from Dolon were conducted independently by six laboratories [6, 15, 64, 66, 68]. Averaged over all available TL/OSL data (six labs) the settlement-average dose to air is equal to 484±75 mGy [5]. The uncertainties of the average values ​​given here correspond to two standard deviations (± 2SD). - Results of individual dose estimations using instrumental ESR method of retrospective dosimetry with human tooth enamel samples show the dose value averaged among 17 inhabitants of the settlement equal to 144 mGy (range 30-350 mGy) [16,50,74-76]. These 17 people lived in the settlement of Dolon for at least one year from the time of the test. Interpretation of the ESR data needs consideration for shielding, behavior, location and migration factors for the inhabitants. These factors are reducing ESR dose in relation to dose to air. According to [5, 14] the mean value of the combination of these factors is 0.28 ± 0.068 for Kazakhstan village. The uncertainties of the average values ​​given here correspond to two standard deviations (± 2SD).As a result, the rough estimate of dose to air based on EPR data is 144 mGy/0.28 = 514 mGy. - Estimates of average dose to air in Dolon for the test on 29.08.1949, derived from the archival exposure rate data with accounting for ^137^Cs soil contamination, are not in contradiction with the estimates of dose to air based on results of TL/OSL measurements in quartz-containing samples from bricks, and on the results of ESR retrospective dosimetry using tooth enamel samples. - Estimations based on available values of exposure rates provide the ranges of external doses to air following the test on 29.07.1955 and the test on 07.08.1962 are as follows: 0.4-0.6 mGy and 0.1-1.5 mGy, respectively.   Conclusion: Taking into account all the data and considerations above, the estimated settlement-average dose to air in Dolon is 500 mGy with the range of 350-650 mGy after the test on 29.08.1949.  The ranges of external doses to air following the test on 29.07.1955 and following the test on 07.08.1962 are as follows: 0.4-0.6 mGy and 0.1-1.5 mGy, respectively. |
| --- |
