# Supplementary material for: External radiation dose reconstruction for settlements near the Semipalatinsk nuclear test site, Kazakhstan, in the international multicenter study: a detailed review and comparative analysis of the initial data
Source: J Radiat Res. 2025 Aug 30;66(5):496–508. doi: 10.1093/jrr/rraf049 (PMC12460053; doi:10.1093/jrr/rraf049)
Supplement: JRRS_D_25_00036_R1_Suppl_Table_9_Revised_No_Hig_rraf049 [file jrrs_d_25_00036_r1_suppl_table_9_revised_no_hig_rraf049.docx]

Supplementary Table 9 (ST 9). Settlement Kainar. Available dose rate data and calculated external doses to air based on these data^*)^ (see List of references in the main part of the paper).

| Date of explosion | Time related to exposure rate estimation, H+h, h | Exposure  Rate | Units | Time of fallout arrival,h | Reference | Calculated dose to air,  mGy |
| --- | --- | --- | --- | --- | --- | --- |
| 24.09.1951 | 3 | 1142 | mR/h | 6.4 | [42,82] | 75 |
| 24.09.1951 | 10 | 0.27 | R/h |  | [43] | 110 |
| 24.09.1951 | 10 | 0.27 | R/h |  | [44] |  |
| 24.09.1951 | 10 | 0.87 | R/h |  | [33] | 350 |
| 24.09.1951 | 24 | 0.094 | R/h |  | [43] | 110 |
| 24.09.1951 | 24 | 0.094 | R/h |  | [35] |  |
| 24.09.1951 | 24 | 0.0713 | R/h |  | [42] | 77 |
| 24.09.1951 | 24 | 0.215 | R/h |  | [40] | 230 |
| 24.09.1951 | 24 | 0.304 | R/h |  | [43] | 330 |
| 24.09.1951 | 24 | 304.3 | mR/h |  | [33] |  |
| 24.09.1951 | 24 | 0.304 | R/h |  | [81] |  |
| 05.10.1954 | 3 | 0.17 | R/h | 3.9 | [42, 33] | 13 |
| 05.10.1954 | 3 | 0.22 | R/h |  | [40] | 17 |
| 05.10.1954 | 24 | 0.014 | R/h |  | [44] | 17 |
| 05.10.1954 | 24 | 0.018 | R/h |  | [19] | 22 |
| 05.10.1954 | 24 | 0.43-1.31 | mR/h |  | [35] | 0.5-1.6 |
| 05.10.1954 | 24 | 0.4 | mR/h |  | [42] | 0.5 |
| 02.08.1955 | 3 | 0.31 | R/h | 4.6 | [33] | 22 |
| 02.08.1955 | 3 | 0.18 | R/h |  | [19] | 13 |
| 02.08.1955 | 24 | 0.026 | R/h |  | [40] | 31 |
| 02.08.1955 | 24 | 0.015 | R/h |  | [44] | 18 |

| ^*)^ Comments to Supplementary Table 9:   - Three tests were identified in relation to fallout in and around Kainar. - It is not clear, what is the origin of exposure rate data, direct measurements or the results of recalculation from unknown time of measurements to the time shown in the Supplementary Table 9. - For two tests (05.10.1954 and 02.08.1955) available archival exposure rate data for Kainar are relatively consistent and show the estimates of settlement-average dose to air in the range of 0.5-22 mGy and 13-31 mGy, for each test respectively. - Test on 24.09.1951 provides the main contribution to external exposure in the range of 75-350 mGy (the average value is 210 mGy) based on the available exposure rate data. - In the case of Kainar village measured levels of the ^137^Cs soil contamination densities were equal to 4100 Bq×m^-2^ in 1989 [26] and 1740±1290 Bq/m^2^ in 2007-2012 [58], which resulted in estimates of external doses to air equal to 130 mGy and 90±67 mGy, respectively. The uncertainties of the average values ​​given here correspond to two standard deviations (± 2SD). - It should be noted that dose value of 90±67 mGy (the dose estimates based on ^137^Cs soil contamination data) are not in contradiction with the range of the dose estimates based on the available exposure rate data for Kainar, namely 75-350 mGy. The uncertainties of the average values ​​given here correspond to two standard deviations (± 2SD).   Conclusion: Summing up all the data and considerations above, the estimated settlement-average external doses to air in Kainar are: (a) 75-350 mGy (average of 210 mGy) after the test on 24.09.1951; (b) 0.5-22 mGy (mean of 11 mGy) after the test on 05.10.1954; (c) 13-31 mGy (mean of 22 mGy) after the test on 02.08.1955. |
| --- |
