# Supplementary material for: External radiation dose reconstruction for settlements near the Semipalatinsk nuclear test site, Kazakhstan, in the international multicenter study: a detailed review and comparative analysis of the initial data
Source: J Radiat Res. 2025 Aug 30;66(5):496–508. doi: 10.1093/jrr/rraf049 (PMC12460053; doi:10.1093/jrr/rraf049)
Supplement: JRRS_D_25_00036_R1_Suppl_Table_10_Revised_No_Hig_rraf049 [file jrrs_d_25_00036_r1_suppl_table_10_revised_no_hig_rraf049.docx]

Supplementary Table 10 (ST 10). Settlement Kanonerka. Available dose rate data and calculated external doses to air based on these data^*)^ (see List of references in the main part of the paper).

| Date of explosion | Time related to exposure rate estimation, H+h, h | | Exposure  Rate | Units | Time of fallout arrival, h | Reference | Calculated dose  to air, mGy |
| --- | --- | --- | --- | --- | --- | --- | --- |
| 29.08.1949 | 24 | 0.24 | | R/h | 3.6 | [29, 32,  43, 81] | 300 |
| 29.08.1949 | 24 | 0.24 | | R/h |  | [33] |  |
| 29.08.1949 | 24 | 0.25 | | R/h |  | [26] | 310 |
| 29.08.1949 | 173 | 25 | | mR/h |  | [33] | 240 |
| 29.07.1955 | 3 | 0.01 | | R/h | 3.9 | [33] | 0.8 |
| 29.07.1955 | 24 | 0.825 | | mR/h |  | [19, 40] | 1.0 |
| 29.07.1955 | 24 | 0.6 | | mR/h |  | [11] | 0.7 |
| 07.08.1962 | 2 | 5 | | mR/h | 16.2 | [29] | 0.14 |
| 07.08.1962 | 24 | 1.8 | | mR/h |  | [31] | 1.5 |
| 07.08.1962 | 24 | 0.485 | | mR/h |  | [32] | 0.4 |
| 07.08.1962 | 24 | 0.65-1.8 | | mR/h |  | [33] | 0.5-1.0 |
| 07.08.1962 | 24 | 0.901 | | mR/h |  | [40] | 0.8 |
| 07.08.1962 | 24 | 1 | | mR/h |  | [18] | 0.85 |
| 07.08.1962 | 48 | 0.385 | | mR/h |  | [33] | 0.7 |
| 07.08.1962 | 216 | 0.035 | | mR/h |  | [18] | 0.3 |
| 07.08.1962 | 264 | 0.02 | | mR/h |  | [33] | 0.2 |
| 07.08.1962 | 504 | 0.012 | | mR/h |  | [33] | 0.3 |
| ^*)^ Comments to Supplementary Table 10.   - Three tests were identified that are related to fallout in and around Kanonerka. - Only one test on 29.08.1949 was significant for external dose estimation to the residents of Kanonerka. - It is not clear what is the origin of the exposure rate data, direct measurements or the results of recalculation from the real time of measurements to the time shown in Supplementary Table 10. - For two tests on 29.07.1955 and 07.08.1962, the available exposure rate data for Kanonerka are consistent and show low estimates of the settlement-average dose to air, less than 1.5 mGy. - The estimates of the settlement-average dose to air after the test on 29.08.1949 based on the available exposure rate data are in the range of 240-310 mGy. - Available value of ^137^Cs soil contamination density for Kanonerka settlement is equal to 4300 Bq×m^-2^ in 1989 [26] allowing to estimate the external dose to air of 190 mGy. - Other results of ^137^Cs soil contamination density related to the settlement of Kanonerka are as follows: the value of mean soil contamination density is 2722 Bq×m^-2^ (range 1730-3700 Bq×m^-2^). The corresponding estimate of the mean external dose in Kanonerka gives the value of 210 (range 130-290 mGy), which is not in contradiction with dose estimates based on exposure rate data. - The estimate of settlement-average dose to air based on TL/OSl measurements in bricks is 240±60 mGy [15]. The uncertainties of the average values ​​given here correspond to two standard deviations (± 2SD). - Results of individual dose estimations using instrumental ESR method of retrospective dosimetry with human tooth enamel samples show the dose value averaged among three inhabitants of the settlement equal to 50 mGy (range 22-74 mGy) [80]. These three people lived in the settlement of Kanonerka for at least one year from the time of the test. Interpretation of the ESR data needs consideration for shielding, behavior, location and migration factors for the inhabitants. These factors are reducing ESR dose in relation to dose to air. According to [5,14] the mean value of the combination of these factors is 0.28 ± 0.068 for Kazakhstan village. The uncertainties of the average values ​​given here correspond to two standard deviations (± 2SD). - As a result, the rough estimate of dose to air based on EPR data is 50 mGy/0.28 = 180 mGy, which is not in contradiction with dose estimates based on the dose rate data, equal to 240-310 mGy, TL/OSL data - 240±60 mGy, and ^137^Cs soil contamination data – 210 mGy (range 130-290 mGy). The uncertainties of the average values ​​given here correspond to two standard deviations (± 2SD).   Conclusion: Summing up all the data and considerations above, the estimated settlement-average dose to air in Kanonerka is 210 mGy with the range of 130-310 mGy (test 29.08.1949). For two other tests (29.07.1955 and 07.08.1962) the available archival exposure rate measurements for Kanonerka provide values of external radiation doses to air in the ranges 0.7-1 mGy and 0.14-1 mGy, respectively. | | | | | | | |
