# Supplementary material for: External radiation dose reconstruction for settlements near the Semipalatinsk nuclear test site, Kazakhstan, in the international multicenter study: a detailed review and comparative analysis of the initial data
Source: J Radiat Res. 2025 Aug 30;66(5):496–508. doi: 10.1093/jrr/rraf049 (PMC12460053; doi:10.1093/jrr/rraf049)
Supplement: JRRS_D_25_00036_R1_Supplementary_Table_11_revised_rraf049 [file jrrs_d_25_00036_r1_supplementary_table_11_revised_rraf049.docx]

Supplementary Table 11 (ST 11). Settlement Karaaul. Available dose rate data and calculated external doses to air based on these data^*)^ (see List of references in the main part of the paper).

| Date of explosion | Time related to exposure rate estimation, H+h, h | Exposure rate | Units | Time of fallout arrival, h | Reference | Calculated dose  to air, mGy |
| --- | --- | --- | --- | --- | --- | --- |
| 12.08.1953 | 24 | 0.475-0.809 | R/h | 3.5 | [42, 18] | 550-940 |
| 12.08.1953 | 24 | 642 | mR/h |  | [43] | 750 |
| 12.08.1953 | 84 | 0.18 | R/h |  | [19] | 870 |
| 12.08.1953 | 84 | 0.18 | R/h |  | [44] |  |
| 12.08.1953 | 84 | 180 | mR/h |  | [26] |  |
|  |  |  |  |  |  |  |
| 12.08.1953 | 218 | 0.03 | R/h |  | [33] | 380 |
| 12.08.1953 | 240 | 0.03 | R/h |  | [40] | 430 |
| 12.08.1953 | 240 | 40.5 | mR/h |  | [32] | 580 |

| ^*)^ Comments to Supplementary Table 11:   - One test on 12.08.1953 was identified in relation to fallout in and around Karaaul. - It is not clear what is the origin of exposure rate data - direct measurements or the results of recalculation from the real time of measurements to the time shown in Supplementary Table 11. - Eight archival exposure rates are available for this test. The range of dose to air estimates in Karaaul derived from those archival exposure rates is 380-940 mGy. - In Karaul the range of the ^137^Cs soil contamination values in Karaaul settlement is 580-4200 Bq×m^-2^ (mean value 2500 Bq×m^-2^) in 2007-2010 [58]. This range corresponds to the range of dose to air in the settlement from 60 mGy to 410 mGy (with mean value of external dose equal to 240 mGy). - Additional information regarding ^137^Cs measurement in Karaaul is available in [26]: the value is 5900 Bq×m^-2^ for 1989 (this is inventory – depth in soil up to 1 m). It corresponds to the estimate of dose to air in the settlement equal to 370 mGy. - So, the estimates of dose to air in the settlement derived from the ^137^Cs measurements in soil, equal in average to 240 mGy (range 60-410 mGy) using [58] and 370 mGy using [26]. It is not in contradiction with the range of dose estimates based on the exposure rates data.   Conclusion:  Summing up all the data and considerations above, mean value of the external dose estimates to air in Karaaul based on the exposure rate data, is equal to 660 mGy (with the range of 380-940 mGy), which is not in contradiction with dose estimates based on ^137^Cs soil contamination data.  Note: To assess external exposure to the residents of Karaaul, it is necessary to consider the evacuation of these residents from the settlement that took place from the date of explosion until 10 days after explosion [18]. |
| --- |
