# Supplementary material for: External radiation dose reconstruction for settlements near the Semipalatinsk nuclear test site, Kazakhstan, in the international multicenter study: a detailed review and comparative analysis of the initial data
Source: J Radiat Res. 2025 Aug 30;66(5):496–508. doi: 10.1093/jrr/rraf049 (PMC12460053; doi:10.1093/jrr/rraf049)
Supplement: JRRS_D_25_00036_R1_Supplementary_Table_12_Revised_rraf049 [file jrrs_d_25_00036_r1_supplementary_table_12_revised_rraf049.docx]

Supplementary Table 12 (ST 12). Settlement Kaskabulak. Available dose rate data and calculated external doses to air based on these data^*)^ (see List of references in the main part of the paper).

| Date of explosion | Time related to exposure rate estimation, H+h, h | Exposure rate | Units | Time of fallout arrival, h | Reference | Calculated dose to air, mGy |
| --- | --- | --- | --- | --- | --- | --- |
| 30.10.1954 | 3 | 0.47 | R/h | 5.8 | [18, 42] | 31 |
| 30.10.1954 | 3.2 | 0.6 | R/h |  | [19] | 45 |
| 30.10.1954 | 24 | 0.054 | R/h |  | [40] | 60 |
| 30.10.1954 | 24 | 0.015 | R/h |  | [33] | 17 |

| ^*)^ Comments to Supplementary Table 12:   - One test on 30.10.1954 was identified in relation to fallout in and around Kaskabulak. - It is not clear from what is the origin of the exposure rate data - direct measurements or the results of recalculation from the real time of measurements to the time shown in Supplementary Table 12). - Four archival exposure rates available for Kaskabulak are consistent. The range of the dose to air estimates in the settlement derived from the archival exposure rates data is 17-60 mGy. - Only three ^137^Cs contemporary measurements in Kaskabulak were found [58]. The range of ^137^Cs soil contamination density is equal to 1133-1588 Bq×m^-2^. It corresponds to the estimates of dose to air in the settlement of 48-76 mGy.   Conclusion: Summing up all the data and considerations above, priority was given to the range of settlement-average dose to air derived from the archival exposure rate measurements. So, the estimated settlement-average dose to air in Kaskabulak using dose exposure rate data is 40 mGy, which is within the range of 17-60 mGy and comparable with the external dose estimations based on ^137^Cs soil contamination data of 48-76 mGy. |
| --- |
