# Supplementary material for: External radiation dose reconstruction for settlements near the Semipalatinsk nuclear test site, Kazakhstan, in the international multicenter study: a detailed review and comparative analysis of the initial data
Source: J Radiat Res. 2025 Aug 30;66(5):496–508. doi: 10.1093/jrr/rraf049 (PMC12460053; doi:10.1093/jrr/rraf049)
Supplement: JRRS_D_25_00036_R1_Suppl_Table_13_Revised_No_Hig_rraf049 [file jrrs_d_25_00036_r1_suppl_table_13_revised_no_hig_rraf049.docx]

Supplementary Table 13 (ST 13). Settlement Korosteli. Available external exposure dose rate data and calculated external doses to air based on these data^*)^ (see List of references in the main part of the paper).

| Date of explosion | Time related to exposure rate estimation, H+h, h | Exposure rate | Units | Time of fallout arrival, h | Reference | Calculated dose to air, mGy |
| --- | --- | --- | --- | --- | --- | --- |
| 29.08.1949 | 3 | 590 | mR/h | 6.1 | [42, 31, 43, 82] | 42 |
| 29.08.1949 | 5 | 200 | mR/h |  | [11] | 34 |
| 29.08.1949 | 24 | 0.263 | R/h |  | [43] | 290 |

| ^*)^ Comments to Supplementary Table 13:  - One test on 29.08.1949 was identified in relation to fallout in and around Korosteli.  -Three archival exposure rate measurements (estimates) are available for Korosteli. They are not consistent. Estimates of the dose to air in the settlement derived from these exposure rate data are in the range from 34 to 290 mGy.  -According to Gordeev et al. [41, 42], the designation of the time point of 24 h related to exposure rate estimation of 0.263 R/h is wrong (possible misprint). Instead, the time of 5 h was recommended as related to exposure dose rate of 0.263 R/h, which results in the corresponding value of external dose equal to 45 mGy. As a result the calculated dose to air based on exposure dose rates, and with accounting for this correction, is within the range of 34-45 mGy for the Korosteli village.  - Only two measurements of ^137^Cs are available in soil samples, 2890 Bq×m^-2^ and 4420 Bq×m^-2^, collected in 1997 from a pasture on the way from the city of Semipalatinsk towards the village of Korosteli [53]. This corresponds to an external dose range in air of 160 mGy to 250 mGy. However, it is unclear to what extent the ^137^Cs measurement in the pasture can be representative of the contamination of the village of Korosteli.  -Map with the trajectory of the radioactive cloud related to 29.08.1949 test [33] shows that the location of Korosteli is relatively far from the axis of the trajectory (48 km). It supports the assumption of low dose to air in Korosteli.  Conclusion: Summing up all the data and considerations above, a priority was given to dose estimations based on exposure rate data, including correction of time point with exposure rate of 0.264 R/h. So, the estimated settlement-average dose to air in Korosteli using dose exposure rate data is 38 mGy, with the range of 34-45 mGy. |
| --- |
