# Supplementary material for: External radiation dose reconstruction for settlements near the Semipalatinsk nuclear test site, Kazakhstan, in the international multicenter study: a detailed review and comparative analysis of the initial data
Source: J Radiat Res. 2025 Aug 30;66(5):496–508. doi: 10.1093/jrr/rraf049 (PMC12460053; doi:10.1093/jrr/rraf049)
Supplement: JRRS_D_25_00036_R1_Supplementary_Table_14_revised_rraf049 [file jrrs_d_25_00036_r1_supplementary_table_14_revised_rraf049.docx]

Supplementary Table 14 (ST 14). Settlement Kundizdi (now Zhurekadir). Available dose rate and calculated external doses to air based on these data^*)^ (see List of references in the main part of the paper).

| Date of explosion | Time of measurement,  H+h, h | Exposure  Rate | Units | Time of fallout arrival, h | Reference | Dose to air,  mGy |
| --- | --- | --- | --- | --- | --- | --- |
| 30.10.1954 | 3 | 0.6 | R/h | 6.7 | [33, 41] | 39 |
| 30.10.1954 | 24 | 0.0495 | R/h |  | [41] | 53 |

| ^*)^ Comments to Supplementary Table 14:   - One test was identified (30.10.1954) in relation to fallout in and around Kundizdi. - Two archival exposure rate data for Kundizdi are consistent. The range of the dose to estimates in the settlement derived from the archival exposure rate measurements is 39-53 mGy. - Results of six ^137^Cs measurements in soil samples for Kundizdi settlement were published in the paper [53]. The range of measured values of ^137^Cs soil contamination density is 247-3606 Bq×m^-2^. It corresponds to the estimates of dose to air in the settlement within a very wide range, from 11 mGy to 166 mGy (more than order of magnitude). It is to note, that locations of soil sampling in relation to the borders of the settlement are unclear. Considering these circumstances, a priority was given to the range of settlement-average dose to air derived from the archival exposure rate data.   Conclusion: Summing up all the data and considerations above, priority was given to the range of settlement-average dose to air based on archival exposure rate data. So, the estimated range of settlement-average dose to air in Kundizdi is 39-53 mGy with the mean value of 46 mGy. |
| --- |
