# Supplementary material for: External radiation dose reconstruction for settlements near the Semipalatinsk nuclear test site, Kazakhstan, in the international multicenter study: a detailed review and comparative analysis of the initial data
Source: J Radiat Res. 2025 Aug 30;66(5):496–508. doi: 10.1093/jrr/rraf049 (PMC12460053; doi:10.1093/jrr/rraf049)
Supplement: JRRS_D_25_00036_R1_Suppl_Table_15_Revised_No_Hig_rraf049 [file jrrs_d_25_00036_r1_suppl_table_15_revised_no_hig_rraf049.docx]

Supplementary Table 15 (ST 15). Settlement Mostik. Available dose rate data and calculated external doses to air based on these data^*)^ (see List of references in the main part of the paper).

| Date of explosion | Time related to exposure rate estimation, H+h, h | Exposure  Rate | Units | Time of fallout arrival, h | Reference | Calculated to air,  mGy |
| --- | --- | --- | --- | --- | --- | --- |
| 29.08.1949 | 24 | 0.085 | R/h | 2.3 | [42, 43] | 120 |
| 29.08.1949 | 24 | 85-107 | mR/h |  | [33, 43] | 120-150 |
| 29.08.1949 | 173 | 0.01 | R/h |  | [43] | 110 |
| 29.08.1949 | 173 | 0.01 | R/h |  | [44] |  |
| 29.08.1949 | 173 | 10 | mR/h |  | [26] |  |
| 07.08.1962 | 2 | 5 | mR/h | 10.8 | [33, 43] | 0.15 |
| 07.08.1962 | 24 | 0.253 | mR/h |  | [33] | 0.24 |

| ^*)^ Comments to Supplementary Table 15:   - Two tests were identified (29.08.1949 and 07.08.1962) in relation to fallout in and around Mostik. - Five exposure rate values available for Mostik for the test on 29.08.1949 are consistent. The range of the dose to air estimates in the settlement derived from the exposure rate data is of 110-150 mGy. - Two archival exposure rate values available for Mostik for the test on 07.08.1962 are consistent. The range of the dose to air estimates in the settlement derived from the archival exposure rate data is of 0.15-0.24 mGy, i.e. less than 1 mGy. - Five ^137^Cs soil contamination density contemporary measurements in Mostik were published in the paper [58]. The range of ^137^Cs soil contamination density values is 1626-2767 Bq×m^-2^. It corresponds to the estimates of dose to air in the settlement equal to 135-230 mGy. - Results of individual dose estimations using instrumental ESR method of retrospective dosimetry with human tooth enamel samples show the dose value averaged among 12 inhabitants of the settlement equal to 53 mGy (range from 16 mGy to 70 mGy) [16, 17, 70, 75, 76, 80]. These 12 people lived in the settlement of Mostik for at least one year from the time of the test. Interpretation of the ESR data needs consideration for shielding, behavior, location and migration factors for the inhabitants. These factors are reducing ESR dose in relation to dose to air. According to [5, 14] the mean value of the combination of these factors is 0.28 ± 0.068 for Kazakhstan village. The uncertainties of the average values ​​given here correspond to two standard deviations (± 2SD). As a result, the rough estimate of dose to air based on ESR data is 53 mGy/0.28 = 190 mGy, which is not in contradiction with dose estimates, based on the ^137^Cs soil contamination data. - The ranges of settlement’s dose to air derived from the archival exposure rate measurements and from ^137^Cs measurements are overlapping. - Priority was given to the range of settlement-average dose to air derived from the exposure rate estimations. At the same time, the range of settlement-average dose to air derived from the exposure rate measurements is worth expanding by raising the upper limit, accounting for the data on ^137^Cs soil contamination density measurements, from 150 mGy to 230 mGy. So, the estimated range of settlement-average dose to air in Mostik is 110-230 mGy.   Conclusion: Summing up all the data and considerations above, the estimated settlement-average dose to air in Mostik is 170 mGy with the range of 110-230 mGy. For the test on 07.08.1962, the estimated value of external dose to air is in the range 0.15-0.24. |
| --- |
