# Supplementary material for: External radiation dose reconstruction for settlements near the Semipalatinsk nuclear test site, Kazakhstan, in the international multicenter study: a detailed review and comparative analysis of the initial data
Source: J Radiat Res. 2025 Aug 30;66(5):496–508. doi: 10.1093/jrr/rraf049 (PMC12460053; doi:10.1093/jrr/rraf049)
Supplement: JRRS_D_25_00036_R1_Supplementary_Table_16_Revised_rraf049 [file jrrs_d_25_00036_r1_supplementary_table_16_revised_rraf049.docx]

Supplementary Table 16 (ST 16). Settlement Novopokrovka. Available dose rate and calculated external doses to air based on these data^*)^ (see List of references in the main part of the paper).

| Date of explosion | Time related to exposure rate estimation, H+h, h | Exposure  Rate | Units | Time of fallout arrival, h | Reference | Calculated dose to air,  mGy |
| --- | --- | --- | --- | --- | --- | --- |
| 29.08.1949 | 3 | 3.4 | mR/h | 4.7 | [43] | 0.26 |
| 29.07.1955 | 3 | 0.01 | R/h | 5.1 | [31] | 0.69 |
| 29.07.1955 | 24 | 0.82 | mR/h |  | [19, 40] | 0.93 |
| 07.08.1962 | 2 | 20 | mR/h | 21.6 | [31, 33] | 0.51 |
| 07.08.1962 | 24 | 1 | mR/h |  | [29, 32] | 0.79 |
| 07.08.1962 | 216 | 0.115 | mR/h |  | [31, 44] | 0.94 |

| *) Comments to Supplementary Table 16:   - Three tests were identified (29.08.1949, 29.07.1955 and 07.08.1962) in relation to fallout in and around Novopokrovka. - For two tests (29.07.1955 and 07.08.1962) the ranges of dose to air estimates in the settlement derived from the exposure rate data are 0.69-0.93 mGy and 0.51-0.94 mGy, respectively, that is less than 1 mGy for each test. - For test on 29.08.1949, the dose rate value is available for Novopokrovka [43]. Considering that the exposure rate is related to the time of 3 hours after explosion (3.4 mR/h at H+3h), it is likely to be an estimate, not a measurement. - Only one ^137^Cs soil contamination density measurement of 810 Bq×m^-2^ in 1989, which was associated with Novopokrovka, was published [26]. It corresponds to the estimate of dose to air in the settlement of 6.8 mGy. Unfortunately, the location of soil sampling in relation to Novopokrovka village was not described and, as a result, is not known. - The NIIRME Registry database [33] contains another measurement of ^137^Cs in soil for Novopokrovka (500 Bq×m^-2^ in 1963 in the soil layer of 0-1 cm), which is close to the level of global fallout in Kazakhstan. This measurement supports an assumption on low ^137^Cs deposition density. Indirectly it confirms a low external dose to air in Novopokrovka. - Map with the trajectory of radioactive cloud from the test on 29.08.1949 [33] shows that the location of Novopokrovka is relatively far from the axis of that trajectory (about 40 km). It also supports the assumption of low dose to air.   Conclusion: Summing up all the data and considerations above, the estimated settlement-average dose to air in Novopokrovka is less than 1 mGy for each test: 0.26 mGy for test 29.08.1949, 0.69-0.93 mGy for test 29.07.1955, and 0.51-0.94 mGy for test 07.08.1962. |
| --- |
