# Supplementary material for: External radiation dose reconstruction for settlements near the Semipalatinsk nuclear test site, Kazakhstan, in the international multicenter study: a detailed review and comparative analysis of the initial data
Source: J Radiat Res. 2025 Aug 30;66(5):496–508. doi: 10.1093/jrr/rraf049 (PMC12460053; doi:10.1093/jrr/rraf049)
Supplement: JRRS_D_25_00036_R1_Supplementary_Table_17_Revised_rraf049 [file jrrs_d_25_00036_r1_supplementary_table_17_revised_rraf049.docx]

Supplementary Table 17 (ST 17). Settlement Sarzhal. Available dose rate data and calculated external doses to air based on these data^*)^ (see List of references in the main part of the paper).

| Date of explosion | Time related to exposure rate estimation,  H+h, h | Exposure rate | Units | Time of fallout  arrival, h | Reference | Calculated dose to air, mGy |
| --- | --- | --- | --- | --- | --- | --- |
| 12.08.1953 | 24 | 1.031-1.096 | R/h | 2.0 | [40] | 1440-1530 |
| 12.08.1953 | 25.7 | 1.19 | R/h |  | [19] | 1800 |
| 12.08.1953 | 25.7 | 1.19 | R/h |  | [44] |  |
| 12.08.1953 | 25.7 | 1200 | mR/h |  | [42] |  |
| 12.08.1953 | 360 | 0.015-0.037 | R/h |  | [40] | 460-1140 |
| 12.08.1953 | 384 | 0.015-0.037 | R/h |  | [33] | 510-1250 |
| 30.10.1954 | 3 | 0.59 | R/h | 5.5 | [44] | 40 |
| 30.10.1954 | 24 | 0.02634 | R/h |  | [33] | 30 |

| ^*)^ Comments to Supplementary Table 17:   - Two tests were identified (12.08.1953 and 30.10.1954) in relation to fallout in and around Sarzhal. - The range of the dose to air estimates in Sarzhal derived from archival exposure rate measurements related to the test on 12.08.1953 is 460-1800 mGy. However, it looks like the exposure rate data assigned to H+24 h and H+25.7h are the theoretical estimates, they correspond to the dose to air estimates of 1440-1800 mGy. While the exposure rate data assigned to H+360h and H+384h are the actual measurements, they correspond to the dose to air estimates in range of 460-1250 mGy. - The range of the dose to air estimates in Sarzhal derived from the exposure rate measurements related to the test on 30.10.1954 is 30-40 mGy. It is substantially less than the estimates of dose to air in Sarzhal for the test on 12.08.1953. - The values of ^137^Cs soil contamination density in Sarzhal ranged from 1100 to 5700 Bq×m^-2^ in 2007-2010 with mean value 4429 Bq×m^-2^ [58]. The mean value ^137^Cs soil contamination density corresponds to the mean external dose to air in the settlement equal to 450 mGy (with the range 112-580 mGy). - Another result of ^137^Cs measurement in single sampling point in Sarzhal settlement was published in [26] with the value of ^137^Cs soil contamination density of 6400 Bq×m^-2^ in 1989. It corresponds to the estimate of dose to air in the settlement equal to 410 mGy. - The estimates of dose to air in the settlement based on ^137^Cs soil contamination density values are: 450 mGy (range 112-580 mGy) from the results of measurements published in [58] and 410 mGy from the results of single point measurement published in [26]. These values are not in contradiction with the dose estimates based on the archival exposure rate measurements (range of 460-1250 mGy).   Conclusion: Summing up all the data above, the priority was given to the range of settlement-average dose to air based on the actual measurements of exposure rates (460-1250 mGy), which is not in contradiction with dose estimates based on ^137^Cs soil contamination data. So, the estimated settlement-average dose to air in Sarzhal is 850 mGy (it is the mean value from the range 460-1250 mGy). The settlement-average dose to air in Sarzhal from the test on 30.10.1954 is 35 mGy (it is the mean value from the range 30-40 mGy).  Note: To assess external exposure to the groups of residents in Sarzhal it is necessary to account for the evacuation of the residents from the settlement which took place before the date of explosion and lasted up to 16 days after the explosion [18]. |
| --- |
