# Supplementary material for: External radiation dose reconstruction for settlements near the Semipalatinsk nuclear test site, Kazakhstan, in the international multicenter study: a detailed review and comparative analysis of the initial data
Source: J Radiat Res. 2025 Aug 30;66(5):496–508. doi: 10.1093/jrr/rraf049 (PMC12460053; doi:10.1093/jrr/rraf049)
Supplement: JRRS_D_25_00036_R1_Supplementary_Table_18_Revised_rraf049 [file jrrs_d_25_00036_r1_supplementary_table_18_revised_rraf049.docx]

Supplementary Table 18 (ST 18). Settlement Zhetizhar (former Semiyarka). Available dose rate data and calculated external doses to air based on these data^*)^ (see List of references in the main part of the paper).

| Date of explosion | Time related to exposure rate estimation, H+h, h | Exposure rate | Units | Time of fallout arrival, h | Reference | Calculated dose to air, mGy |
| --- | --- | --- | --- | --- | --- | --- |
| 07.08.1962 | 7 | 1 | mR/h | 7,4 | [32,42] | 0.25 |
| 07.08.1962 | 24 | 1.4 | mR/h |  | [32] | 1.5 |
| 07.08.1962 | 24 | 0.5 | mR/h |  | [29] | 0.52 |
| 07.08.1962 | 25.5 | 5 | mR/h |  | [32] | 5.6 |
| 07.08.1962 | 33 | 1 | mR/h |  | [32] | 1.5 |
| 07.08.1962 | 504 | 0.03 | mR/h |  | [32] | 1.0 |
| 07.08.1962 | 552 | 0.025 | mR/h |  | [32] | 0.93 |

| ^*)^ Comments to Supplementary Table 18:   - Only one test was identified in relation to fallout in and around Zhetizhar (former Semiyarka). - Seven exposure rates records related to fallout in and around Zhetizhar (former Semiyarka) were identified. - It is not clear what is the origin of exposure rate data -direct measurements or the results of recalculation from the real time of measurements to the time shown in Supplementary Table 18). - There are no available data on ^137^Cs soil contamination density measurements in relation to Zhetizhar (former Semiyarka).   Conclusion: Summing up all the data and considerations above, the estimated settlement-average dose to air in Zhetizhar (former Semiyarka) is 3 mGy with the range of 0.25-5.6 mGy after the test on 07.08.1962. |
| --- |
