# Supplementary material for: External radiation dose reconstruction for settlements near the Semipalatinsk nuclear test site, Kazakhstan, in the international multicenter study: a detailed review and comparative analysis of the initial data
Source: J Radiat Res. 2025 Aug 30;66(5):496–508. doi: 10.1093/jrr/rraf049 (PMC12460053; doi:10.1093/jrr/rraf049)
Supplement: JRRS_D_25_00036_R1_Supplementary_Table_19_revised_rraf049 [file jrrs_d_25_00036_r1_supplementary_table_19_revised_rraf049.docx]

Supplementary Table 19 (ST 19). Settlement Znamenka. Available dose rate data and calculated external doses to air based on these data^*)^ (see List of references in the main part of the paper).

| Date of explosion | Time related to exposure rate estimation, H+h, h | Exposure  Rate | Units | Time of fallout arrival, h | Reference | Calculated dose to air, mGy |
| --- | --- | --- | --- | --- | --- | --- |
| 12.08.1953 | 24 | 9.0-28.0 | mR/h | 2.0 | [44] | 13-39 |
| 12.08.1953 | 24 | 0.009-0.028 | R/h |  | [40] |  |
| 12.08.1953 | 24 | 0.7-2.06 | R/h |  | [33] | 980-2900 |
| 12.08.1953 | 37.6 | 1.2 | R/h |  | [19] | 2800 |
| 30.10.1954 | 24 | 0.187-0.815 | mR/h | 4.6 | [33] | 0.22-0.96 |
| 16.03.1956 | 1.7 | 882 | mR/h | 3.8 | [42] | 27 |
| 16.03.1956 | 3 | 208 | mR/h |  | [33] | 16 |
| 16.03.1956 | 24 | 22.9 | mR/h |  | [32] | 28 |
| 16.03.1956 | 24 | 17.7-22.9 | mR/h |  | [40] | 22-28 |
| 16.03.1956 | 720 | 0.3 | mR/h |  | [32] | 19 |
| 24.08.1956 | 24 | 50.2-75.4 | mR/h | 2.1 | [29, 32] | 75-110 |
| 24.08.1956 | 24 | 75.4 | mR/h |  | [40] | 110 |
| 24.08.1956 | 24 | 17.8-75.4 | mR/h |  | [33] | 27-110 |
| 24.08.1956 | 720 | 0.3 | mR/h |  | [32] | 23 |
| 24.08.1956 | 720 | 0.3 | mR/h |  | [44] |  |
| 07.08.1962 | 24 | 1.1 | mR/h | 15 | [33] | 1.0 |
| 07. 08.1962 | 24 | 0.387-0.425 | mR/h |  | [44] | 0.34-0.37 |
| 07. 08.1962 | 48 | 0.15 | mR/h |  | [19] | 0.28 |
| 07. 08.1962 | 48 | 0.185 | mR/h |  | [32] | 0.35 |
| 07. 08.1962 | 504 | 0.01 | mR/h |  | [31] | 0.28 |
| 25.09.1962 | 24 | 4.48 | mR/h | 4.6 | [18] | 5.3 |
| 25.09.1962 | 24 | 0.61 | mR/h |  | [81] | 0.71 |
| 25.09.1962 | 24 | 0.61-4.744 | mR/h |  | [33] | 0.71-5.6 |
| 25.09.1962 | 24 | 0.61-8.0 | mR/h |  | [44] | 0.71-9.4 |
| 25.09.1962 | 30 | 0.62 | mR/h |  | [32] | 0.95 |
| 25.09.1962 | 72 | 0.28 | mR/h |  | [32] | 1.0 |
| 15.01.1965 | 24 | 0.05 | R/h | 3.8 | [35, 44] | 62 |

| *) Comments to Supplementary Table 19:   - Seven tests were identified (12.08.1953, 30.10.1954, 16.03.1956, 24.08.1956, 07.08.1962, 25.09.1962, and 15.01.1965) related to fallout in and around Znamenka settlement. - It is not clear -what is the origin of the exposure rate data, direct measurements or the results of recalculation from the real time of measurements to the time shown in Supplementary Table 19. - For the test on 12.08.1953, the available exposure rate values are contradictory. For the other six tests the data are largely consistent. - For two tests on 30.10.1954 and 07.08.1962, the exposure rate results in a low range of dose to air estimates, for each test the dose range is below 1 mGy. - For the test on 16.03.1956, the range of estimated external dose to air is 16-28 mGy. - For the test on 24.08.1956, the range of estimated external dose to air is 23-110 mGy. - For the test on 25.09.1962, the range of estimated external dose to air is 1-9 mGy. - For the test on 15.01.1965, the external dose to air estimate is 62 mGy. - For the test on 12.08.1953, the range of calculated external dose to air on the base of two exposure rate values from [44,40], is 13-39 mGy. For two other available exposure rate values the external dose provided the external dose values equal to 980 mGy and 2900 mGy. The difference between the high dose values of 980-2900 mGy based on these two dose rates records and much lower dose values of 13-39 mGy based on two other dose rates values is about 100 times. It should be noted that very different values of the exposure rate are related to the same time (H+24h): 9-28 mR/h versus 700-2060 mR/h. - In this regard, it will be useful to compare contradictive dose values ​​calculated from the exposure dose rates with the estimates calculated from ^137^Cs soil contamination density. Results of the ^137^Cs soil contamination density measurements in Znamenka were 2000 Bq×m^-2^ in 1989 [26] and 873 Bq×m^-2^ in 1995 [51,52]. They correspond to the dose to air equal to 75 mGy and 22 mGy, respectively. As far as ^137^Cs soil contamination data reflects the sum of the dose contributions from all tests listed in the Supplementary Table 19, results of this comparison should be considered as conservative. Considering that estimates of dose to air derived from the ^137^Cs measurements in Znamenka (22-75 mGy), even being conservative, are much lower in comparison with estimates based on the exposure rate after the test on 12.08.1953 resulting in the dose to air with the range of 980-2900 mGy, it should be concluded that dose values of 980-2900 mGy are not confirmed and can be considered as erroneous due to a possible misprint in the exposure rate initial data. - Conclusion: Summing up all the data and considerations above it will be reasonable to accept external dose estimations on the base of exposure rate data, without accounting for two erroneous exposure rate values, which were discussed above. So, the estimated settlement-average external doses to air based on exposure rate data in Znamenka are as follows: - For the test on 12.08.1953, the external dose is 26 mGy with the range of 13-39 mGy. - For two tests on 30.10.1954 and 07.08.1962, the external doses are in the ranges 0.22-0.96 mGy and 0.28-1 mGy, respectively. - For the test on 16.03.1956, the external dose is 22 mGy with the range of 16-28 mGy. - For the test on 24.08.1956, the external dose is 67 mGy with the range of 23-110 mGy. - For the test on 25.09.1962, external dose is 5 mGy with the range of 0.7-9.4 mGy. - For the test of 15.01.1965, external dose is 62 mGy. |
| --- |
