# Supplementary material for: External radiation dose reconstruction for settlements near the Semipalatinsk nuclear test site, Kazakhstan, in the international multicenter study: a detailed review and comparative analysis of the initial data
Source: J Radiat Res. 2025 Aug 30;66(5):496–508. doi: 10.1093/jrr/rraf049 (PMC12460053; doi:10.1093/jrr/rraf049)
Supplement: 8_JRRS_D_25_00036_R1_Supplement_revised_No_Highlight_2025_rraf049 [file 8_jrrs_d_25_00036_r1_supplement_revised_no_highlight_2025_rraf049.docx]

**SUPPLEMENT**

Annexes containing additional data and discussions are presented below.

Annexes are followed by 19 Supplementary Tables (Supplementary Tables 1-19).

**Annexes with additional data and discussions.**

*Annex 1: To estimated external dose values, which are presented in the Table 1 of the main text of the paper.*

According to the results of our estimations, which are presented in the Table 1 of the main text of the paper, the considered settlements can be conditionally divided into three groups, in accordance with the estimated values ​​of accumulated doses of external irradiation to the air (from the highest doses to the lowest). These groups, indicated below as A, B, C, are as follows.

Group A (high doses values, 10 settlements). Sarzhal: 850 mGy (range 460-1250 mGy) from the test on 12.08.1953, and 35 mGy (range30-40 mGy) from the test 30.10.1954; Karaaul: 660 mGy (range 380-940 mGy) from the test 12.08.1953; Dolon: 500 mGy (range 350-650 mGy) from the test 29.08.1949; Cheremushka: 430 mGy (range 63-800 mGy); Bodene: 260 mGy (range 200-320 mGy) from the test 29.08.1949; Chagan: 220 mGy (range 190-240 mGy) from the test 29.08.1949, and 70 mGy from the test 15.01.1965; Kainar: 210 mGy (range 75-350 mGy) from the test 24.09.1951, and 11 mGy (range 0.5-22 mGy) from the test 05.10.1954, and 22 mGy (range 13-31 mGy) from the test 02.08.1955; Akbulak: 210 mGy from the test 24.09.1951; Kanonerka: 210 mGy (range 130-310 mGy) from the test 29.08.1949; Mostik: 170 mGy (range 110-230 mGy) from the test 29.08.1949.

As it follows from Table 1, additional inputs to irradiation of each of the settlements, listed in item A) above, are less than 1.6 mGy from each of other considered tests.

Group B (middle doses group, 4 settlements). Kundizdi: 46 mGy (range 39-53 mGy) from the tets 30.10. 1954; Kaskabulak: 40 mGy (range 17-60 mGy) from the test 30.10.1954; Korosteli: 38 mGy (range 34-45 mGy) from the test 29.08.1949; Znamenka: 67 mGy (range 23-110 mGy) from the test 24.08.1956, and 62 mGy from the test 15.01.1965, and 26 mGy (range 13-39 mGy) from the test 12.08.1953, and 22 mGy (range 16-28 mGy) from the test 16.03.1956, and 5 mGy (range 0.7-9.4 mGy) from the test 25.09.1962.

As it follows from Table 1, additional inputs to irradiation of each of the settlements, listed in item B) above, are ≤ 1 mGy from each of other considered tests.

“Group C (lowest dose group, 4 settlements, the lowest doses of radiation can be used in radiation epidemiological studies for comparison with higher doses of radiation). Bolshaya Vladimirovka: (1 -1.4 mGy) from the test 29.08.1949, and (0.8-1 mGy) from the test 29.07.1955; Novopokrovka: (0.69-0.93 mGy) from the test 29.07.1955, and (0.51-0.94 mGy) from the test 07.08.1962, and 0.26 mGy from the test 29.08.1949; Belokamenka: (0.35-0.56 mGy) from the test 07.08.1962, and (0.4-0.5 mGy) from the test 29.08.1949, and (0.08-0.1 mGy) from the test 29.07.1955; Zhetizhar: 3 mGy (range 0.25-5.6 mGy) from the test 07.08.1962.”

*Annex 2: To the comparisons between settlement-average values of accumulated external radiation doses in the air, calculated by authors, with previously published values of accumulated external radiation doses in the air.*

Table 1 of the main text of the article presents the results of a comparison of the external irradiation doses in the air obtained in our study with the values ​​of external irradiation doses that were published in available publications [45, 58, 85, 86]. As follows from Table 1, these comparisons are not available for all populated areas and not for all tests. Unfortunately, due to the lack of information on external radiation doses in air, comparisons were made only for 12 settlements (Akbulak, Belokamenka, Bolshaya Vladimirovka, Bodene, Cheremushka, Dolon, Kainar, Kanonerka, Karaul, Mostik, Sarzhal, and Znamenka) - out of 18 settlements considered in our work and listed in Table 1. For the same reason, comparisons were made only for traces from four tests (08.29.1949, 09.24.1951, 08.12.1953, and 08.24.56) - out of 13 tests considered in our work and listed in Table 1.

Nevertheless, the obtained results of comparison allowed to conditionally divide the compared values ​​of external radiation doses in the air into the following three groups, which are discussed below.

1) Group 1 (the values ​​of external irradiation doses in the air obtained in our study correspond to the values ​​of doses available in the cited literature, or the ranges of the compared dose values ​​are overlap):

- The village of Bodene - our estimate is 260 mGy (range 200-320 mGy) vs. 200 mGy [85] (for the test on 29.08.1949);

- The village of Dolon - our estimate is 500 mGy (range 350-650 mGy) vs. 600 mGy (range 350-910 mGy) [45, 89] (for the test on 29.08.1949);

- The settlement of Kainar - our estimate is 210 mGy (range 75-350 mGy) vs. 114-870 mGy [85] and 360 mGy [86] (for the test on September 24, 1951);

- The village of Karaaul - our estimate is 660 mGy (range 380-940 mGy) vs. 890-1300 mGy [85] and 120-380 mGy [58] (for the test on August 12, 1953);

- The village of Mostik - our estimate is 170 mGy (range 110-230 mGy) vs. 180 mGy [85] and 170 mGy [86] (for the test on August 29, 1949);

- The village of Sarzhal - our estimate is 850 mGy (range 460-1250 mGy) vs. 930 mGy [85] and 210-530 mGy [58] (for the test on 12.08.1953);

- The village of Znamenka - our estimate is 67 mGy (range 23-110 mGy) vs. 80 mGy [85] and 120 mGy [86] (for the test on 24.08.1956).

It should be noted here, that compared estimates of external radiation doses in the air, presented in Table 1 and included in Group 1, are based on different initial archival data and on different methods of retrospective dosimetry. Nevertheless, the dose values ​​are consistent with each other. This can be considered as an argument in favor of the high degree of validity of the presented values ​​of external radiation doses.

2) Group 2 (the values ​​of external irradiation doses in air obtained in our study do not correspond to the dose values ​​available in some publications, or the ranges of the compared dose values ​​do not overlap):

- The village of Dolon - our estimate of 500 mGy (range 350-650 mGy) vs. 1950 mGy [85] and 1950 mGy [86] (for the test of 29.08.1949).

Notes - in the work [85] the presented dose value is relates to the axis of the radioactive trace, but not to the settlement; in the work [86] the exact location of dose rate, which was used in the calculations, is not indicated in relation to the location of the settlement.

- The village of Cheremushka - our estimate is 430 mGy (range 63-800 mGy) vs. 1700 mGy [85] and 2970 [86] (for the test on 08.29.1949).

Notes - in the work [85] the presented dose value is relates to the axis of the radioactive trace, but not to the settlement; in the work [86] the exact location of dose rate, which was used in the calculations, is not indicated in relation to the location of the settlement.

-The village of Kanonerka - our estimate is 210 mGy (range 130-310 mGy) vs. 500 mGy [85] and 350 mGy [86] (for the test on 29.08.1949).

Notes – the exact location of dose value in relation to the considered settlement is not indicated in the work [85]; in the work [86] the exact location of dose rate, which was used in the calculations, is not indicated in relation to the location of the settlement.

-The village of Karaaul - our estimate is 660 mGy (range 380-940 mGy) vs. 1150 mGy [86] (for the test on 12.08.1953).

Note - in the work [86] the exact location of dose rate, which was used in the calculations, is not indicated in relation to the location of the settlement;

- The village of Sarzhal – our estimate is 850 mGy (range 460-1250 mGy) vs. 2090 mGy [86] (for the test of 12.08.1953).

Note – in the work [86] the exact location of dose rate, which was used in the calculations, is not indicated in relation to the location of the settlement;

It is necessary here to discuss the importance of such parameter for calculating external radiation doses as the location of dose rate measurement.

For example, the values ​​of external radiation dose in the air for village of Dolon presented in publications [85,86] significantly exceed our dose estimates and the dose values, which are ​​consistent with our estimates, and published in [45,89] (see Table 1 in the main text). The reasons for this excess are noted and discussed in the comments to Table 1 in the main text and in Table 8 in the “Supplementary Data and Materials”. Thus, work [85] provides the estimate of the dose in the air corresponding to the axis of the trajectory of the radioactive cloud trace near the village of Dolon, but not for the settlement itself, which is located at a distance of several kilometers from the axis of this trajectory [14, 55]. Meanwhile, the trace of radioactive fallout in the vicinity of Dolon after the test of 29.08.1949 has a significant gradient towards the village, both in terms of the dose rate and in terms of soil contamination by 137Cs [14, 55], which must be taken into account when calculating external radiation dose corresponding to the location of the settlement [5], which, apparently, was not taken into account either in publication [85] or in publication [86]. In addition, publication [86] does not indicate the exact source of the data on dose rate used, nor does not indicate the location of dose rate measurements in relation to the settlement.

Thus, indicating and taking into account the location of absorbed dose rate in relation to the considered settlement is of principle importance when calculating external radiation dose in the considered settlement. This is noted in the comments to Table 1 of the main text and to Tables 2-19 of the “Supplementary Data and Materials”. Indeed, this is so, since during nuclear tests the dose rate changes not only along the trajectory of the radioactive cloud’s trace, but also in directions transverse to this axis. Therefore, failure to take this factor into account can significantly affect the validity of the external radiation dose value attributed to the settlement located at a distance from the place of dose rate measurement – ​​away from the axis of the radioactive trace.

3)Group 3 (the compared absorbed doses do not coincide, but their values ​​are quite close to each other and belong to the range of the lowest doses of external irradiation in the air - less than 5 mGy):

-The village of Belokamenka - our estimate is 0.4-0.5 mGy vs. 0.9 mGy [85] (for the test on 29.08.1949);

-The village of B. Vladimirovka - our estimate is 1-1.4 mGy vs. 1.8 mGy [85] (for the test on 29.08.1949).

*Annex 3: Regarding features of retrospective dosimetry methods as applied to the data obtained in this work.*

In our work the methods of calculational retrospective dosimetry were used in external dose estimations, and the available results of instrumental TL/ESR retrospective dosimetry were used for comparisons with calculated dose values. In this regard, it is useful to note the essential features of retrospective dosimetry methods that distinguish them from conventional methods of dosimetry. .

Unlike conventional (common) methods of dosimetry, when it is possible to determine fairly accurately the current conditions that affect the formation of the radiation dose, methods of retrospective dosimetry takes place with radiation exposure events that took place many years ago. When reconstructing the dose-forming conditions that took place many years ago, one has to deal with archival data that have various degrees of completeness and may also have gaps in the necessary information. That is why this work was devoted to a critical comparative analysis and selection of the most realistic archival and published data, which are the initial ones for the retrospective assessment of radiation doses.

*Annex 4: Regarding peculiarities of comparison of various archival sources of information on exposure rate values ​​and data on soil contamination by ^137^Cs.*

A comparison of different information sources on the exposure rate values and ^137^Cs soil contamination data is useful for revealing contradictions between various archival records or published data, which are required for the retrospective assessment of accumulated doses of external irradiation to air after the tests conducted at the SNTS many years ago. This is because the available archival and published data on the exposure rates and/or ^137^Cs soil contamination after the tests are not always complete, and sometimes the data reliability is in question, as it follows from the comments to the Table 1in the main text of the paper and to the Tables 1-19 in the “Supplementary Data and Materials”. For example, a comparison of the available exposure rates related to different time points after the test shows that in some cases it was necessary to decide - whether these data are the results of direct measurements, or they are a product of theoretical calculations, or there were simple misprints [43] in the archival records [33, 41]. Moreover, available information about locations of the exposure rates’ measurements is not always clear. It makes the understanding difficult - whether the measurements are linked to the locations within a study settlement, or they related to locations just near the traces of radioactive clouds [14]. The traces of radioactive fallout can be very narrow [14, 55, 81], so the exposure rate in a settlement located at some distance from the trajectory of the radioactive cloud can differ significantly from the exposure rate more close to trajectory [14, 33, 55]. In these cases information on spatial distributions of soil contamination densities by long-lived radionuclides can be useful for refining of radioactive fallout trajectories’ locations [5, 6, 13, 14, 51-60, 81].

*Annex 5: Rerding features of interpretation of dose estimates based on ^137^Cs soil contamination data density, which reflect the sum of the contributions from all tests associated with considered settlement*

It should be noted, that when the measurements of the ^137^Cs soil contamination density were applied to our dose estimates for some settlement, the used contamination level reflected the sum of the contributions from all tests associated with considered settlement. This raises the question of how the total ^137^Cs deposition density was distributed among the ^137^Cs soil contamination densities from individual nuclear tests. Obviously, separating the contributions of individual tests to the total contamination is problematic in general terms.

However, for our study, it is appropriate to consider the specific situations for different settlements among 18 settlements, which were included to the study. The corresponding considerations are as follows.

-For nine settlements this question does not arise because: a) for three settlements (Belokamenka, Bolshaya Vladimirovka, and Semiyarka) ^137^Cs contamination data are absent; b) six settlements are associated with only one test (Akbulak, Bodene, Karaul, Kazkabulak, Korosteli, and Kundidzi).

-For seven settlements (Chagan, Cheremushka, Dolon, Kainar, Kanonerka, Mostik, Sarzhal), whose exposure was due to several tests, there were situations when one of these tests significantly exceeded the others in terms of radiation exposure, which is confirmed by the available data on the exposure dose rates after the tests. Thus, the contributions of other tests for these seven settlements can be considered much smaller compared to the prevailing contribution from one test, and the measured ^137^Cs soil contamination densities can be interpreted as relating mainly to the test with the largest contribution to the radiation dose.

-Two settlements (Novopokrovka and Znamenka) were contaminated as a result of several tests, among which it was difficult to indicate the most important one. It is worth noting that in these two cases the values of ^137^Cs soil contamination density were significantly lower than in the other seven settlements mentioned above, for which one test could be identified as the primary one. In cases where no single test could be identified as the major contributor to the radiation dose, a conservative approach was used, in which the ^137^Cs soil contamination density was associated with each of the considered tests, related to the settlement.

In addition, it should be noted here, that calculations of local external radiation doses corresponded to each local point in the territory where soil samples were taken are meaningless, as far as these point calculations cannot characterize the settlement as a whole. It is the average radiation dose for the settlement that is necessary, since the irradiation of population is averaging due to the movement of residents of the settlement across the territory. The usual practice is the estimation of settlement average radiation dose based on averaging of contamination values ​​measured at different sampling points, as recommended in the relevant methodological guidelines [18,41].

For example, in the case of the village of Dolon, were the reported number of soil sampling points with information about GPS coordinates of these points is equal to 89 [14], the value of average soil contamination density is 5870 Bq×m^-2^ (range 4070-7670 Bq×m^-2^). The corresponding estimate of the average external radiation dose based on ^137^Cs soil contamination in Dolon gives the value of 500 mGy (range 350-650 mGy) - see Table 1 in the main text of the paper and Supplementary Table 8 in “Supplementary Data and Materials”.

However, it is to note, that if the number of points with available results of ^137^Cs soil contamination density is small for considered settlement, then the average value of radioactive contamination should be considered as only approximate and less reliable, and in this case it is advisable to indicate the range of values ​​of radioactive contamination [18,41].

*Annex 6: Regarding features and complementarity of various methods of retrospective dosimetry in application to the reconstruction of external radiation doses after nuclear tests.*

Results of different instrumental TL/OSL and ESR retrospective dosimetry methods are considered as helpful information for validation of calculated external dose estimates [50, 64-68, 70,73-80]. Information available on radioactive soil contamination density (for example, by long-lived ^137^Cs [51-54, 56-60]) is assumed to be a useful source of complimentary data for the assessment of external doses in the study settlements as well [62].

Meanwhile, the method for estimating air dose based on TL/OSL measurements of quartz inclusions in bricks and the method for estimating air dose based on ^137^Cs measurements are based on different principles. Therefore, from a general point of view, the possibility of comparing dose estimates obtained by different methods may appear to be difficult. However, both of these methods were successfully applied for retrospective assessment of external doses in air around SNTS and after the Chernobyl accident [5, 15, 83, 84]. Moreover, comparison of the results shows a reliable correlation between the values of average doses in air for populated areas obtained by different methods, including TL/OSL dosimetry with quartz containing samples [50, 84].

An important part of retrospective TL/OSL dosimetry method is taking into account the conversion factor from the dose in a brick to the dose in air [15, 83, 84]. The correction for self-shielding is calculated using the Monte Carlo method taking into account the geometry of the building (wall), the position and orientation of the quartz-containing sample in it. In addition the calculation of correction factor needs accounting for the peculiarities of ^137^Cs contamination of the area around the location of considered building. For this purpose, selecting of territories around buildings with available data about locations of soil sampling points, and information about possible changes in soil contamination with time due to natural processes or human interventions, is necessary. All of these items are important parts of dose estimations methodology based on TL/OSL measurements [14, 15, 83, 84]. At same time it should be pointed here that the similar methodology is applied when taking radioactive soil samples near locations of interest: it is also require documentation of sampling locations, careful consideration when selecting soil sampling sites and considering possible changes in soil contamination with time due to natural processes or human interventions [13].

The application of this methodology resulted the overall agreement in retrospective estimations of external doses, obtained by several laboratories experienced in the use of quartz for dosimetry, which indicate that the method is robust when applied in the settlements around SNTS and downwind Chernobyl NPP [15, 83, 84], with reliable correlation between the values of settlement-average external doses obtained by TL/OSL method and by dose calculations based on ^137^Cs soil contamination [84].

**Supplementary Tables titles**

Supplementary Table 1 (ST 1). List of 18 study settlements with available information on the dates,

heights, yields, settlements’ coordinates and locations of the tests, for which the radioactive fallout were

considered as possible sources of external irradiation of the population (data from [25, 27, 28, 33-39], and

from database of NIIRME State Registry [10, 18, 33] are presented - see List of references in the main

part of the paper).

Supplementary Table 2 (ST 2). Settlement Akbulak. Available exposure dose rate data,

^137^Cs soil contamination density, and calculated external doses to air based on these data*)

(see List of references in the main part of the paper).

Supplementary Table 3 (ST 3). Settlement Belokamenka. Available exposure dose rate data and

calculated external doses to air based on these data*)

(see List of references in the main part of the paper).

Supplementary Table 4 (ST 4). Settlement Bolshaya Vladimirovka (now Beskaragay). Available

exposure dose rate data and calculated external doses to air based on these data*)

(see List of references in the main part of the paper).

Supplementary Table 5 (ST 5). Settlement Bodene. Available exposure dose rate data and calculated

external doses to air based on these data*)

(see List of references in the main part of the paper).

Supplementary Table 6 (ST 6). Settlement Chagan. Available exposure dose rate data and calculated

external doses to air based on these data*)

(see List of references in the main part of the paper).

Supplementary Table 7 (ST 7). Settlement Cheremuska. Available exposure dose rate data and calculated

external doses to air based on these data*)

(see List of references in the main part of the paper).

Supplementary Table 8 (ST 8). Settlement Dolon. Available dose rate data and calculated external doses to air

based on these data*)

(see List of references in the main part of the paper).

Supplementary Table 9 (ST 9). Settlement Kainar. Available dose rate data and calculated external doses to air

based on these data*)

(see List of references in the main part of the paper).

Supplementary Table 10 (ST 10). Settlement Kanonerka. Available dose rate data and calculated external doses to

air based on these data*)

(see List of references in the main part of the paper).

Supplementary Table 11 (ST 11). Settlement Karaaul. Available dose rate data and calculated external doses to air

based on these data*)

(see List of references in the main part of the paper).

Supplementary Table 12 (ST 12). Settlement Kaskabulak. Available dose rate data and calculated external doses to

air based on these data*)

(see List of references in the main part of the paper).

Supplementary Table 13 (ST 13). Settlement Korosteli. Available external exposure dose rate data and calculated

external doses to air based on these data*)

(see List of references in the main part of the paper).

Supplementary Table 14 (ST 14). Settlement Kundizdi (now Zhurekadir). Available dose rate and calculated

external doses to air based on these data*)

(see List of references in the main part of the paper).

Supplementary Table 15 (ST 15). Settlement Mostik. Available dose rate data and calculated external doses to air

based on these data*)

(see List of references in the main part of the paper).

Supplementary Table 16 (ST 16). Settlement Novopokrovka. Available dose rate and calculated external doses to

air based on these data*)

(see List of references in the main part of the paper).

Supplementary Table 17 (ST 17). Settlement Sarzhal. Available dose rate data and calculated external doses to air

based on these data*)

(see List of references in the main part of the paper).

Supplementary Table 18 (ST 18). Settlement Zhetizhar (former Semiyarka). Available dose rate data and calculated

external doses to air based on these data*)

(see List of references in the main part of the paper).

Supplementary Table 19 (ST 19). Settlement Znamenka. Available dose rate data and calculated external doses to

air based on these data*)

(see List of references in the main part of the paper).
